# Increased T cell trafficking as adjunct therapy for HIV-1

**DOI:** 10.1371/journal.pcbi.1006028

**Published:** 2018-03-02

**Authors:** Helen R. Fryer, Steven M. Wolinsky, Angela R. McLean

**Affiliations:** 1 Institute for Emerging Infections, Department of Zoology, University of Oxford, The Peter Medawar Building for Pathogen Research, South Parks Road, Oxford, United Kingdom; 2 Division of Infectious Diseases, Northwestern University Feinberg School of Medicine, Chicago, Illinois, United States of America; University of Texas at Austin, UNITED STATES

## Abstract

Although antiretroviral drug therapy suppresses human immunodeficiency virus-type 1 (HIV-1) to undetectable levels in the blood of treated individuals, reservoirs of replication competent HIV-1 endure. Upon cessation of antiretroviral therapy, the reservoir usually allows outgrowth of virus and approaches to targeting the reservoir have had limited success. Ongoing cycles of viral replication in regions with low drug penetration contribute to this persistence. Here, we use a mathematical model to illustrate a new approach to eliminating the part of the reservoir attributable to persistent replication in drug sanctuaries. Reducing the residency time of CD4 T cells in drug sanctuaries renders ongoing replication unsustainable in those sanctuaries. We hypothesize that, in combination with antiretroviral drugs, a strategy to orchestrate CD4 T cell trafficking could contribute to a functional cure for HIV-1 infection.

## Author summary

Despite the success of potent antiretroviral therapy in suppressing the amount of virus in peripheral blood for long periods of time, a reservoir of infectious virus persists in CD4 T cells, implying the need for long-term treatment. Strategies to control and ultimately eliminate the viral reservoir within specific tissue compartments will need to target virus that persists in both a long-lived reservoir of infectious virus in CD4 T cells as well as low-levels of viral replication that continues despite antiretroviral drug therapy. Using a mathematical model, we describe a hypothetical new therapeutic approach to eliminating HIV-1 persistence in these ‘drug sanctuaries’. Specifically, we show that therapy that increases the rate that the target cells for HIV-1 flow through drug sanctuaries could stop continuous cycles of replication. Used in combination with antiretroviral treatment, such a therapy could contribute to a functional cure for HIV-1.

## Introduction

Despite the success of HIV-1 therapies in reducing the concentration of virus in the bloodstream [1], a long-lived reservoir of infectious virus persists in CD4 T cells [2–6] and perhaps other cell types [7]. Although most of the proviral DNA within CD4 T cells is not able to replicate [8], replication-competent virus can persist in long-lived resting memory CD4 T cells in a quiescent state [4,5,9–11]. These latently infected cells, which are replenished through proliferation [12] or new infection can release infectious virus when reactivated [4,5]. HIV-1 can also be derived from ongoing cycles of replication of CD4 T cells in tissue compartments where antiretroviral drugs have difficulty reaching–the so called drug sanctuaries [13–15]–and viral particles produced from infected CD4 T helper follicular cells that are captured and presented on the follicular dendritic cell network [16].

An effective cure strategy will need to target both the latent and active viral reservoir. Thus far, strategies to eliminate the viral reservoir have focused on early initiation of antiretroviral therapy (ART) [17], increasing the administered amount of current antiretroviral drugs [18] or manipulation of cellular and viral transcription factors that eliminate transcriptional or post-transcriptional blocks [19–23]. Curative strategies focused on the activation of dormant virus that would lead to its destruction via host immune or viral cytopathic effects have not led to a reduction in the number of infected cells, however [8,24–29].

The enrichment of infected cells within secondary lymphoid tissue and lymph nodes suggest a critical role for these anatomical sites in sheltering persistently infected cells during therapy [7,13,30]. HIV-1 RNA is particularly abundant in germinal centers within lymphoid tissue [31–33]. Regulatory mechanisms that prevent self-reactivity in the germinal center keep this microenvironment relatively safe from cytotoxic CD8 T cells and natural killer (NK) cells that target and destroy infected cells [34–38], thereby giving this unique tissue compartment immune privilege. This impaired protective immunity along with natural epigenetic silencing mechanisms and poor drug penetration allows the virus continue to replicate [39]. As neither antiretroviral drugs, nor immune cells are able to fully abate ongoing cycle of replication in lymphoid tissue, other strategies need to be sought.

A novel approach is to influence how CD4 T cells traffic between lymphoid tissue and peripheral sites where antiretroviral drugs effectively penetrate. Immune cell migration plays a critical role in immunity. Trafficking is affected by the cell’s differentiation status and activation state and is orchestrated by chemokines, integrins and selectins, as well as cues from the microenvironment [40–42]. Humanized monoclonal antibodies that influence immune cell traffic are currently being explored for the treatment of inflammatory disorders [43,44] and cancerous tumors [45]. A therapeutic strategy that could influence the traffic of CD4 T cells so that HIV-1 infected cells migrate more quickly out of drug sanctuaries could also control persistent replication.

Here, we develop a mathematical framework that defines how manipulating CD4 T cell trafficking could influence HIV-1 control. By boosting the traffic of CD4 T cells harboring virus out of drug sanctuaries to regions where antiretroviral drugs effectively penetrate, the virus cannot continue to replenish the persistent viral reservoir. Our analyses show that if the rate of trafficking of infected CD4 T cells exceeds a certain critical threshold, ongoing cycles of viral replication in drug sanctuaries become unsustainable. This strategy points towards a novel adjunct strategy to the treatment of HIV-1 infection and a promising new approach to a functional cure.

## Methods

### A mathematical model of ongoing replication in a drug sanctuary

**Fig 1:**
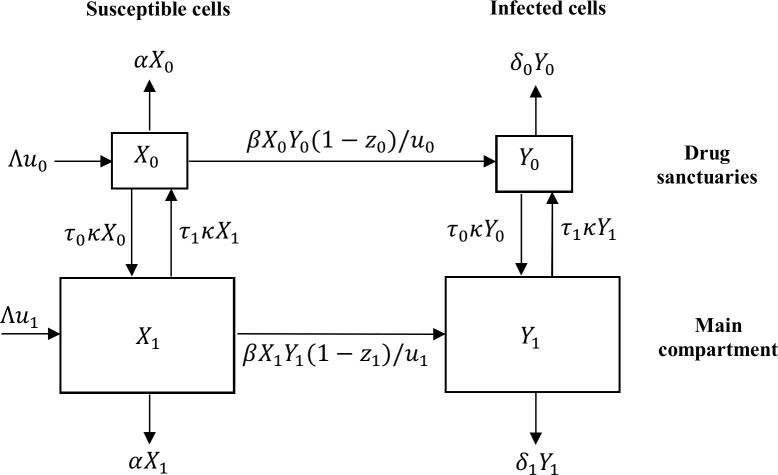
A mathematical model of ongoing replication in drug sanctuaries. The model tracks the number of susceptible and infected cells in each of two spatial compartments, which can differ in size and in the effectiveness of antiretroviral therapy. Within each spatial compartment, infected cells can only transmit infection to other cells within the same spatial compartment, but a fraction of transmission is blocked by antiretroviral drugs. The model includes trafficking of cells between the two compartments, the rate of which can be changed using a trafficking therapy. The model also includes cell turnover.

The spatial and dynamic mathematical model [13,46,47] (model 1; see Fig 1 and Eqs 1–4) has two spatial compartments that differ in size and in the effectiveness of antiretroviral therapy: the drug sanctuaries (i = 0) and the main compartment (i = 1). In the drug sanctuaries, the effectiveness of antiretroviral therapy (the proportional decrease in infection probability due to drugs) is insufficient to block ongoing cycles of HIV-1 replication. Although many drug sanctuaries could exist within the body, for simplicity we model the sum of these sanctuaries as a single compartment. Our threshold results are robust to this assumption. Notably, the drug sanctuaries could include small regions within lymphoid tissue [13]. It is clear that these regions must be very small because HIV-1 infected cells in lymphoid tissue, as well as HIV-1 RNA in plasma, declines by many orders of magnitude on antiretroviral therapy [15]. The main compartment represents regions of the body that include peripheral blood and the majority of the lymphoid tissue where antiretroviral therapy is sufficiently effective that sustained viral replication is not possible.

Within each of the two spatial compartments there is homogenous mixing that facilitates the spread of HIV-1 between infected and uninfected CD4 T cells. Though the risk of a cell becoming infected within each compartment comes only from that compartment, there is also trafficking of CD4 T cells between compartments [48]. Principally, we consider the impact of an envisaged therapy that increases the rate at which CD4 T cells traffic between these two compartments. We also investigate the impact of combining such a “trafficking therapy” with therapy that improves immunological control inside drug sanctuaries (“immune therapy”). These spatial dynamics are built upon a standard compartmental model of the impact of antiretroviral drug therapy on the infection of CD4 T cells [49].

A fraction (*u*_*i*_) of the body’s CD4 T cells are assumed to be in each compartment (*u*_0_ + *u*_1_ = 1) and the production rate of uninfected CD4 T cells in the body is apportioned between the drug sanctuaries (*Λu*_0_ day^-1^) and the main compartment (*Λu*_1_ day^-1^). In the absence of antiretroviral therapy, susceptible cells (*X*_*i*_) within each compartment become infected cells (*Y*_*i*_) at a rate equal to the product of the density of infected cells in the same compartment (*Y*_*i*_/*u*_*i*_ day^-1^) and the transmission parameter *β*. This assumption ensures that the basic reproductive number of each compartment, if it were isolated (i.e. in the absence of traffic of cells between compartments), is not directly related to the size of the compartment. Antiretroviral therapy blocks a fraction, *z*_*i*_, of these infections. The parameter *z*_*i*_ is allowed to vary between compartments, with *z*_0_ ≪ *z*_1_ ≈ 1, so that antiretroviral therapy is highly effective in the main compartment. In the absence of trafficking therapy, cells travel from compartment *i* to the other compartment at a per cell rate of *τ*_*i*_ day^-1^. The constraint, *τ*_0_*u*_0_ = *τ*_1_*u*_1_, ensures no change in compartment size in the absence of infection. The envisaged trafficking therapy that we focus on acts to increase trafficking in both directions by a factor κ. In adaptations of this model discussed later, we also model trafficking therapy that independently manipulates the rate that cells flow into or out of drugs sanctuaries. Infected cells in compartment *i* are cleared at a per cell rate of δ_i_ day^-1^. These rates are free to vary between compartments to account for the possibility that drug sanctuaries may also be subject to impaired infected cell clearance rates (*δ*_0_ < *δ*_1_).

In the presence of immune therapy directed at the drug sanctuaries, the cell clearance rate in the drug sanctuaries can be increased. In both compartments, susceptible cells are cleared at a per cell rate of α day^-1^. The resulting set of coupled, non-linear ordinary differential equations describing the change in the number of susceptible cells (Eqs 1 and 2) and infected cells (Eqs 3 and 4) in the drug sanctuaries and main compartment over time is provided below.

$$dX_{0}/dt=\Lambda u_{0}-\beta X_{0}Y_{0}\left(1-z_{0}\right)/u_{0}-\left(\alpha +\tau_{0}\kappa \right)X_{0}+\tau_{1}\kappa X_{1}$$ (Eq 1)

$$dX_{1}/dt=\Lambda u_{1}-\beta X_{1}Y_{1}\left(1-z_{1}\right)/u_{1}-\left(\alpha +\tau_{1}\kappa \right)X_{1}+\tau_{0}\kappa X_{0}$$ (Eq 2)

$$dY_{0}/dt=\beta X_{0}Y_{0}\left(1-z_{0}\right)/u_{0}-\left(\delta_{0}+\tau_{0}\kappa \right)Y_{0}+\tau_{1}\kappa Y_{1}$$ (Eq 3)

$$dY_{1}/dt=\beta X_{1}Y_{1}\left(1-z_{1}\right)/u_{1}-\left(\delta_{1}+\tau_{1}\kappa \right)Y_{1}+\tau_{0}\kappa Y_{0}$$ (Eq 4)

## Results

### There is a threshold condition on the CD4 T cell trafficking rate that allows ongoing cycles of replication in drug sanctuaries

Our mathematical model reveals a threshold condition in which HIV-1 infection is dependent upon the rate that CD4 T cells traffic between compartments (S1 Text). Assuming that the drug sanctuaries are small in size compared to the main compartment, viral replication in the drug sanctuaries is only sustainable when the rate of trafficking of infected CD4 T cells between the drug sanctuaries and the main compartment is below a critical threshold value. Should infected CD4 T cells traffic from the drug sanctuaries into the main compartment faster than the critical threshold rate, the number of secondarily infected cells that arise and remain in the drug sanctuaries would fall below one, and ongoing replication in the sanctuaries would become unsustainable. That is, the basic reproductive number in the drug sanctuaries would be less than one and ongoing replication could not persist in the long-term.

**Fig 2:**
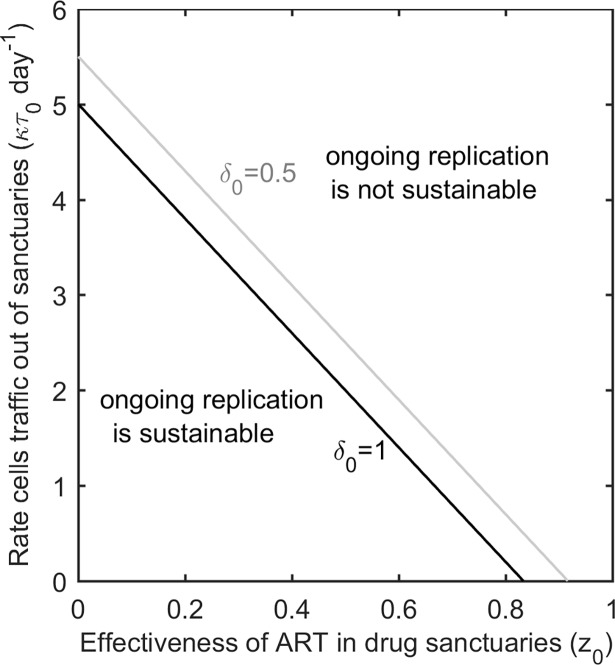
The threshold for ongoing replication is dependent upon several factors including the rate of CD4 T cell trafficking. The grey line plots the relationship between the per cell rate that CD4 T cells traffic out of drug sanctuaries (κτ_0_ day^-1^) and the effectiveness of antiretroviral therapy in the drug sanctuaries (z_0_), under the assumption that the drug sanctuaries are also immune sanctuaries (δ_0_ = 0.5 day^-1^,δ_1_ = 1 day^-1^). Boosting immune control in the sanctuaries so that there are equal infected cell clearance rates in each compartment (δ_0_ = δ_1_ = 1 day^-1^) acts additively with boosting cell trafficking rates so that the threshold for sustainable replication is decreased (black line). This figure reveals that antiretroviral therapy, trafficking therapy and immune therapy could all work in synergy to halt ongoing replication in drug sanctuaries. Guided by clinical findings, these plots assume that antiretroviral therapy is very effective in the main compartment (z_1_ = 0.97) and drug sanctuaries are very small in size compared to the main compartment.

Fig 2 illustrates the threshold for ongoing replication as a function of the rate at which CD4 T cells move between compartments and the effectiveness of antiretroviral therapy in the drug sanctuaries (see S1 Text for derivation). Here, antiretroviral drug therapy is assumed to be highly effective in the main compartment (*z*_1_ = 0.97) and the drug sanctuaries are small in size compared to the main compartment (see S1 Fig for threshold analysis for larger sanctuaries). There is a negative relationship between the effectiveness of antiretroviral therapy in the drug sanctuaries and the rate of cell trafficking between compartments at the threshold for ongoing replication. Even perfect drug sanctuaries (*z*_0_ = 0) can only support ongoing replication provided the rate that CD4 T cells traffic through them is below a critical threshold. When immune control in the sanctuary is boosted, the critical threshold for trafficking is lower, that is, stopping ongoing replication in drug sanctuaries becomes easier.

### Increases in CD4 T cell trafficking could halt ongoing replication

**Fig 3:**
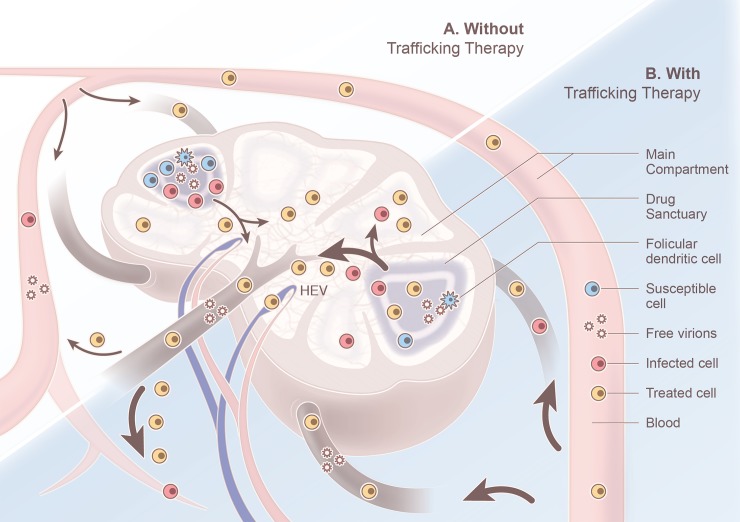
The predicted impact of ‘trafficking therapy’ on ongoing replication in a lymph node. In the absence of therapy that promotes cell trafficking (top left), trafficking of CD4 T cells in and out of the drug sanctuaries is slow enough to allow ongoing cycles of replication. The sanctuaries include small regions within lymph nodes (purple region). The effectiveness of antiretroviral therapy is assumed to be high in other regions, referred to as the main compartment, including elsewhere in lymph nodes (pale region) and in the blood. In the main compartment, continuous cycles of replication are unsustainable. When trafficking therapy increases the trafficking rate above the critical threshold (bottom right), CD4 T cells move more rapidly through the lymphatic system, including between the drug sanctuaries and the main compartment. The egress of infected CD4 T cells from the drug sanctuaries lowers their density in this spatial compartment. As a result, fewer virus particles are produced in the drug sanctuaries. If trafficking is fast enough, the lower density of virus particles and infected cells in the drug sanctuaries combine to ensure that ongoing cycles of infection, either through cell-to-cell infection or free virus, is not sustainable.

In demonstrating that ongoing cycles of replication in drug sanctuaries require CD4 T cell trafficking between compartments to be below a critical threshold, our model suggests a novel approach to HIV-1 control. It predicts that therapy designed to increase the trafficking of CD4 T cells into and out of drug sanctuaries above a threshold rate could halt ongoing cycles of replication. Although a ‘trafficking therapy’ of this sort would affect infected and uninfected cells equally, the mechanism through which it would work is by decreasing the mean residence time of infected cells in drug sanctuaries, moving them into the main compartment where drug concentrations are sufficient to block new rounds of infection with HIV-1 (Fig 3).

**Fig 4:**
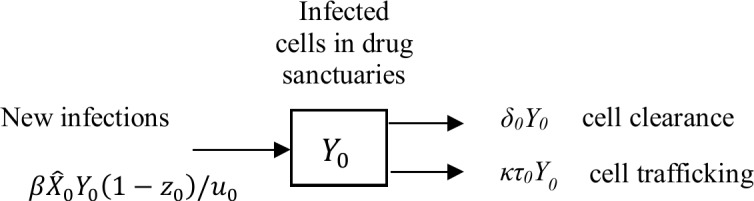
The impact of trafficking on ongoing replication can be understood by focusing on the dynamics concerning the drug sanctuaries. The effective reproductive number can be understood by focusing on just the dynamics concerning infected cell numbers in the drug sanctuaries (Y_0_) when the drug sanctuaries hold only a small fraction of the body’s CD4 T cells. Under this assumption the net traffic of infected cells out of the drug sanctuaries is approximately κτ_0_Y_0_ day^-1^ (see S1 Text for details). The dynamics of infected cells in the drug sanctuaries will therefore be governed by a single influx rate (new infections at rate $\beta {\hat{X}}_{0}(1-z_{0})/u_{0}$ day^-1^) and two efflux rates (cell clearance at rate δ_0_ Y_0_ day^-1^ and cell egress at rate κτ_0_Y_0_ day^-1^).

This can be understood by evaluating, *R*, the effective reproductive number for the system in the presence of therapy, that is, the average number of secondary infected cells generated by one primary infected cell in a given population of target cells. The formal derivation of *R* is presented in S1 Text. Under the simplifying assumption that the CD4 T cell population in the drug sanctuaries (at a given value ${\hat{X}}_{0}$) is very small compared to that in the main compartment, the effective reproductive number (Eq 5) can be understood by focusing on just the dynamics concerning the drug sanctuaries (Fig 4).

$$R=\frac{\beta {\hat{X}}_{0}(1-z_{0})}{u_{0}(\delta_{0}+\kappa \tau_{0})}$$ (Eq 5)

Notice that the effective reproductive number is equal to the rate at which one infected cell infects others ($\beta {\hat{X}}_{0}(1-z_{0})/u_{0}$ day^-1^), multiplied by the mean residence time of infected cells in drug sanctuaries (1/(*δ*_0_ + *κτ*_0_) days). With this expression, it is clear that the effective reproductive number can be reduced by either: increasing the rate that cells traffic through drug sanctuaries (higher *κ*); reducing the number of susceptible cells (target cells) in drug sanctuaries (lower ${\hat{X}}_{0}$); increasing the effectiveness of antiretroviral therapy in drug sanctuaries (higher *z*_0_); or boosting immune control and thus increasing the clearance rate of infected cells in drug sanctuaries (higher *δ*_0_).

The immune system is a highly diverse, complex system and any mathematical model must make simplifying assumptions. The result we present here is robust to any refinements that maintain the core processes drawn in Fig 4. Should the average time that any infected cell spends within the drug sanctuaries be shorter than the average time required for infection of one other uninfected cell, continuous rounds of infection in drug sanctuaries are not sustainable.

**Fig 5:**
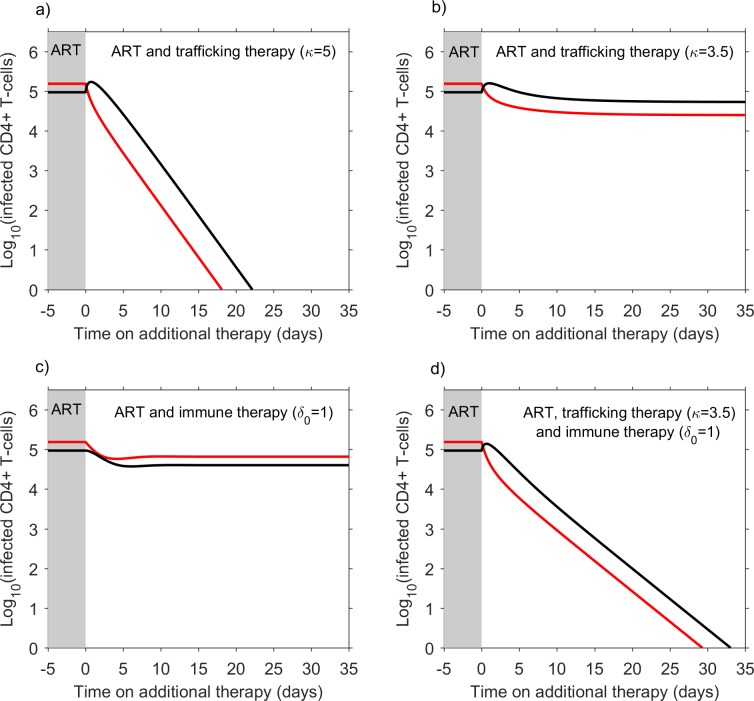
Addition of trafficking therapy can halt ongoing replication in drug sanctuaries. These figures show model predictions of the impact of therapeutic interventions on infected cell numbers in the drug sanctuaries (red lines) and main compartment (black lines). In each figure, prior to time 0 (grey shaded area), the host is only taking antiretroviral therapy (ART). At this stage, the effectiveness of antiretroviral therapy is high in the main compartment of the body (z_1_ = 0.97), but lower in the drug sanctuaries (z_0_ = 0.6). Furthermore, the per cell rate that cells traffic between compartments (governed by parameter *τ*_0_ = 0.5 day^-1^) and the per cell rate that infected cells are cleared from the drug sanctuaries (δ_0_ = 0.5 day^-1^) are slow enough to allow ongoing cycles of replication to persist in the drug sanctuaries. At time 0, additional therapy that either increases the cell trafficking rate (a and b), increases the cell clearance rate of infected cells from the drug sanctuaries (c), or both (d) is applied. In a), the trafficking rate is increased sufficiently (κ = 5) that ongoing viral replication is no longer sustainable. In b), the trafficking rate is increased to a level (κ = 3.5) just below the critical threshold and infected cell numbers decline to a new, lower equilibrium in the drug sanctuaries, but in the main compartment they increase. In c), the per cell clearance rate of infected cells in the drug sanctuaries is increased to the same level as in the main compartment (δ_0_ = 1 day^-1^). Here, the assumption that the effective drug concentration is highly impaired in the drug sanctuaries results in infected cell numbers declining to a new equilibrium. In d) both the per cell trafficking rate and the per cell clearance rate are increased to levels (κ = 3.5 and δ_0_ = 1 day^-1^) which independently would not stop ongoing replication (b and c), but, in combination, do so. All parameter values used in these calculations are provided in S1 Table.

Fig 5 shows model predictions of the impact of changes to the trafficking rate or enhanced immune control upon the dynamics of susceptible and infected CD4 T cells in patients taking antiretroviral therapy. In these simulations, prior to time 0, infected cell numbers under antiretroviral therapy are assumed to have reached an equilibrium state due to continuous replication in drug sanctuaries. After time 0, trafficking therapy, immune therapy or both are applied. Fig 5A shows that if the CD4 T cell trafficking rate is increased above the defined critical threshold there is a transient increase in infected cells in the main compartment as cells are washed out of the drug sanctuaries into the main compartment. Following this, infected cell counts in both compartments decline as replication becomes unsustainable in the drug sanctuaries.

If CD4 T cell trafficking is increased, but to a rate that is still below the critical threshold, the resulting infected cell number depends upon the balance between two effects: first any increase in incidence fueled by a net influx of susceptible cells into the drug sanctuaries (see S2 Text for discussion); and second, faster removal of infected cells from drug sanctuaries. If the trafficking rate is increased to a level just below the critical threshold, infected cell numbers decline to a new, lower equilibrium in the drug sanctuaries, but in the main compartment they increase (Fig 5B). Increasing the clearance of infected cells within drug sanctuaries acts synergistically with increased trafficking of cells out of drug sanctuaries.

Immune therapy that increases the rate of infected-cell clearance in the drug sanctuaries is another approach to stopping ongoing replication and could be encouraged through enhancing the function or penetration of CD8 T cells [50,51], natural killer (NK) cells or innate immunity [52]. But unless clearance rates in the sanctuaries can be boosted beyond the rates observed in the main compartment, this approach alone is likely to be insufficient to halt persistent replication in sanctuaries where the effectiveness of antiretroviral therapy is also very low (Fig 5C). However, trafficking therapy and immune therapy can be expected to act synergistically so that treatments that combine a sub-threshold trafficking therapy and a sub-threshold immune therapy could be sufficient to halt replication (Fig 5D). Improving the penetration of antiretroviral drugs into sanctuaries is a third approach to tackling persistent replication that could work in synergy with these approaches (Fig 2).

### A robust result that holds under different biological assumptions

The human immune response is a complex system composed of multiple cell types operating in many parts of the body. This model is a major simplification of that system aimed at understanding the features most salient to the question in hand–namely how to halt ongoing replication in drug sanctuaries. We reiterate that any modelled therapy that leads to each infected cell in a drug sanctuary giving rise to less than one further infected cell will lead to a modelled cure.

To explore the implications of some of the most pertinent additional complexities we present additional models in the Supporting Information, as follows. In S2 Text (Model 2) we model trafficking therapy that does not directly affect population sizes in each compartment. In S3 Text (Model 3) we model the impact of trafficking therapy that can independently change the rate that cells flow into or out of drugs sanctuaries. In S4 Text we explore the role of free virus under homogenous mixing (Model 4) or spatial heterogeneity (Model 5). In S5 Text (Model 6) we explore the impact of including cells that carry their treatment status with them as they move from the main compartment into the drug sanctuary. Our results remain robust to all these additional complexities.

Model 5 further highlights that any intervention that increases the trafficking rate of free virus, in addition to, or instead of, CD4 T cells, is capable of halting ongoing replication in drug sanctuaries. This is one putative mechanism for how antibodies that bind α_4_β_7_ –an integrin involved in gut homing [53] and demonstrated to integrate into the envelope of HIV-1 [54]–have led to significantly improved control in simian immunodeficiency virus (SIV) infected monkeys [55] (S6 Text). Furthermore, Model 3 shows that just reducing the inflow of CD4 T cells into, or increasing the outflow of CD4 T cells from drug sanctuaries, can also be sufficient to halt persistent replication. This explicitly highlights a fundamental concept of our study: that any intervention that reduces the average residency time of infected CD4 T cells in the drug sanctuaries can halt persistent replication in these regions.

## Discussion

We developed a mathematical model that demonstrates that even a perfect drug sanctuary can only support ongoing replication if infected CD4 T cells traffic through it slowly enough. That is, only anatomical sites that, to a certain degree, are isolated in terms of CD4 T cell mixing have the potential to act as drug sanctuaries. Because persistent replication in drug sanctuaries contributes to the maintenance of the HIV-1 reservoir, our finding opens up an entirely new approach to eliminating this source of virus through the regulation of the trafficking of CD4 T cells to lymphoid tissue.

Increased CD4 T cell trafficking could eliminate persistent replication by promoting infected cells out of drug sanctuaries and into regions with clinically effective drug concentrations where the virus they produce cannot infect uninfected cells. As the average number of secondary infections arising from each infected cell is reduced, ongoing cycles of viral replication no longer remain sustainable. Thus, a novel way to address the problem of concentrations of drugs that are low is to promote the trafficking of infected cells away from drug sanctuaries to anatomical regions where antiretroviral drugs penetrate effectively.

Here we have demonstrated the theoretical potential of using trafficking therapy to stop ongoing replication in drug sanctuaries. There are several practical challenges to the development of safe and effective drugs that can achieve this, however. While this strategy is not restricted to any particular tissue that might harbour a sanctuary, no single therapeutic agent would be expected to usefully impact trafficking to all drug sanctuary sites. By regulating a receptor required for trafficking into one particular tissue, CD4 T-cells could be redistributed to another tissue, where drug sanctuaries also exist. As we have demonstrated (see Eq 5), this could increase the effective reproductive number in these sanctuaries and add to the challenge of HIV-1 clearance from them.

Even within a particular tissue, cells may respond differently to trafficking signals owing to the expression of distinct homing receptors and their differentiation status and activation state [40–42]. Whereas effector memory and central memory T cells recirculate between blood, lymphoid tissue, and lymph, resident memory T cells do not circulate through the blood [56]. Thus, functional changes are associated with T cell migration. Although the existence of some cell types within a drug sanctuary that are unresponsive or less responsive to trafficking signals does not preclude an effective outcome of trafficking therapy, it does make it more difficult. The outcome of trafficking therapy would depend upon the fraction of cells of each type, the degree to which each of their trafficking patterns can be manipulated and the degree to which these different cell types mix together. Ultimately, if different cells types within a sanctuary mix homogeneously, and if the average residency time of infected cells in a drug sanctuary (across all cell types) can be sufficiently reduced, replication can still be rendered unsustainable.

Despite these challenges, targeting just the most important cell types in sanctuary sites that contribute most significantly to persistent infection could still prove worthwhile. By significantly reducing the size of the reservoir, but not necessarily eliminating it, trafficking therapy could be used alongside other therapies or in pursuit of a functional cure (see S6 Text for comments). For instance, gut associated lymphoid tissue is an important site of persistent HIV-1 [7] and the trafficking of immune cells to the gastrointestinal tract can be reduced by integrin inhibitors (vedolizumab and natalizumab) that are routinely used for the treatment of Crohn’s disease and ulcerative colitis [43,57]. These drugs consist of monoclonal antibodies that selectively target the α_4_β_7_ integrin receptor, expressed on immune cells. When used to treat simian immunodeficiency virus-infected monkeys, vedolizumab reduced the amount of virus detected in the blood [55]. Under the hypothesis presented here, the anti- α_4_β_7_ antibody likely helped to control simian immunodeficiency virus infection after antiretroviral drug cessation by encouraging CD4 T cells to traffic away from gut-associated lymphoid tissue, to other tissue sites that have high concentrations of antiretroviral drugs.

Trafficking of immune cells into and out of lymph nodes is essential for immune surveillance of foreign invaders. CD4 T cells have a preference to home to germinal centers once they are activated [58] and follicular helper T cells only circulate amongst the lymph follicles [59]. As immune privileged sites for the activation and selection of B cell responses, the germinal center is outside the scope of cytotoxic CD8 T cells [37,38] or natural killer cells. Accordingly, germinal centers are plausible foci for persistent viral replication [7,31] and offer particular challenges in regards to the use of trafficking therapy.

Because productive HIV-1 infection predominantly occurs in activated CD4 T cells [60], it is implicit that HIV-1 infected cells are less permissive to egress cues compared to uninfected CD4 T cells that normally travel through the germinal center. This degree of difference would significantly reduce the impact of trafficking therapy compared with our model predictions that rely on the assumption that infected and uninfected CD4 T cells respond equally to trafficking therapy. Hypothetical mechanistic approaches to orchestrating T cell trafficking to germinal centers (S7 Text) would involve manipulation of pathways that are central to the immune response; for example S1P-S1PR1-mediated egress of immune cells from lymph nodes into efferent lymphatics [41,61]. In chronic infection, virus-specific CD8 T cells are largely excluded from the germinal center [37,38] or are functionally impaired within it [35]. Thus, a potentially effective adjunctive strategy for control of the viral reservoir would be to restore the ability of these cells to kill infected cells by blocking the PD-1/PD-L1 inhibitory pathway [51] or allow entry of functional antigen-specific T cells by directly engineering CD8 T cells to express CXCR5 [50]

Whether adjunct therapy that targets ongoing replication in drug sanctuaries could ever achieve a full or functional cure will clearly depend upon the relative importance of ongoing replication compared to other sources that contribute to the HIV-1 reservoir in treated patients [12,13,62]. It is currently unknown whether ongoing replication accounts for the majority or a minority of the persistent pool of replication competent HIV-1 and whether alternative sources, including latent infection and homeostatic proliferation of latently infected cells, would be self-sustaining in the absence of ongoing replication that continuously tops up the reservoir. If the pools of virus are self-sustaining, drugs that targets these alternative sources [19] will need to be included in any comprehensive therapeutic strategy to control or clear the viral reservoir.

Arguably, the concept of cell trafficking as therapy is a counterintuitive one because it opposes traditional ideas about the role of mixing in increasing infection rates [63]. Since antiretroviral therapy is highly effective throughout most of the body, including the peripheral blood, drug sanctuaries in HIV-1 infection are akin to small islands of ongoing replication in a sea of highly effective drug concentrations. In such a situation, it is intuitively appealing that increased trafficking of CD4 T cells could reduce their residency time in drug sanctuaries and contribute to a functional cure for HIV-1 infection.

## Supporting information

S1 Text Derivations for the effective reproductive number, basic reproductive number and threshold for ongoing replication for model 1. (PDF)

S2 Text The impact of a trafficking therapy on viral dynamics. (PDF)

S3 Text Model adaptations show that reducing the inflow of CD4+ T-cells into, or increasing the outflow of CD4+ T-cells out of drug sanctuaries can stop persistent replication. (PDF)

S4 Text Infection via free virus does not influence the findings of our study. (PDF)

S5 Text If cells carry their treatment status with them as they migrate, there is still a threshold on the pace of CD4+ T-cell trafficking that can support ongoing replication in drug sanctuaries. (PDF)

S6 Text α_4_β_7_ antibody therapy and a functional cure for HIV-1. (PDF)

S7 Text Manipulating the trafficking of CD4 T-cells to germinal centres. (PDF)

S1 Fig The threshold for ongoing replication is dependent upon several factors including the rate of CD4+ T-cell trafficking and the size of the drug sanctuaries. (PDF)

S1 Table Parameters for model 1. (PDF)
