# Supplementary material for: Increased T cell trafficking as adjunct therapy for HIV-1
Source: PLoS Comput Biol. 2018 Mar 2;14(3):e1006028. doi: 10.1371/journal.pcbi.1006028 (PMC5864072; doi:10.1371/journal.pcbi.1006028)
Supplement: S1 Text — (PDF) [file pcbi.1006028.s001.pdf]

S1 Text. Derivations for the effective reproductive number, basic reproductive number and threshold for ongoing replication for model 1.

To investigate the behaviour of model 1, we calculated several related analytic expressions. First, the effective reproductive number, *R*, defined as the average number of secondary infected cells generated by one primary infected cell in a given population of susceptible cells. This metric does not specify the conditions of the population, for example the fraction of the population that are susceptible or receiving treatment. Second, the basic reproductive number in the absence of therapy (R_0), defined as the average number of secondary infected cells generated by one primary infected cell in a wholly untreated and uninfected population of susceptible cells. Third, the basic reproductive number in the presence of therapy ($R_{0,T}$), defined as the average number of secondary infected cells generated by one primary infection in a wholly uninfected population that is receiving treatment. By evaluating $R_{0,T} > 1$, an analytic expression for the threshold for ongoing replication in the presence of therapy is also derived. Derivation of these expressions begins by calculating the next generation matrix that relates to the model equations (equations 1.1-1.4). These are a set of four coupled ordinary differential equations, described in more detail in the main text.

Model 1 equations

$$\frac{dX_0}{dt} = \Lambda u_0 - \beta X_0 Y_0 (1 - z_0)/u_0 - \alpha X_0 - \kappa \tau_0 X_0 + \kappa \tau_1 X_1 \quad \{1.1\}$$

$$\frac{dX_1}{dt} = \Lambda u_1 - \beta X_1 Y_1 (1 - z_1)/u_1 - \alpha X_1 - \kappa \tau_1 X_1 + \kappa \tau_0 X_0 \quad \{1.2\}$$

$$\frac{dY_0}{dt} = \beta X_0 Y_0 (1 - z_0)/u_0 - \delta_0 Y_0 - \kappa \tau_0 Y_0 + \kappa \tau_1 Y_1 \quad \{1.3\}$$

$$\frac{dY_1}{dt} = \beta X_1 Y_1 (1 - z_1)/u_1 - \delta_1 Y_1 - \kappa \tau_1 Y_1 + \kappa \tau_0 Y_0 \quad \{1.4\}$$

$$\tau_0 u_0 = \tau_1 u_1 \quad \{1.5\}$$

The next generation matrix

To calculate the effective reproductive number (S1 reference), the first step is to formulate the next generation matrix, $\mathbf{K}$. This can be determined by defining terms which represent the 'gains' into (new infections: G_0 and G_1) and 'losses' from (movement of cells between compartments or clearance: L_0 and L_1) the infected categories.

$$\text{gains to } Y_0 : G_0 = \beta X_0 Y_0 (1 - z_0)/u_0 \quad \{1.6\}$$

$$\text{gains to } Y_1 : G_1 = \beta X_1 Y_1 (1 - z_1)/u_1 \quad \{1.7\}$$

$$\text{losses to } Y_0 : L_0 = (\delta_0 + \kappa\tau_0) Y_0 - \kappa\tau_1 Y_1 \quad \{1.8\}$$

$$\text{losses to } Y_1 : L_1 = -\kappa\tau_0 Y_0 + (\delta_1 + \kappa\tau_1) Y_1 \quad \{1.9\}$$

Jacobian matrices **F** and **H** (the elements of which are partial derivatives with respective to each of the infected categories of the gains and losses terms) are then evaluated at the specific position $X_0 = \hat{X}_0$, $X_1 = \hat{X}_1$, $Y_0 = \hat{Y}_0$, $Y_1 = \hat{Y}_1$.

$$\mathbf{F} = \begin{bmatrix} \frac{\partial G_0}{\partial Y_0} & \frac{\partial G_0}{\partial Y_1} \\ \frac{\partial G_1}{\partial Y_0} & \frac{\partial G_1}{\partial Y_1} \end{bmatrix}_{\hat{X}_0, \hat{X}_1, \hat{Y}_0, \hat{Y}_1} = \begin{bmatrix} \beta \hat{X}_0 (1 - z_0)/u_0 & 0 \\ 0 & \beta \hat{X}_1 (1 - z_1)/u_1 \end{bmatrix} \quad \{1.10\}$$

$$\mathbf{H} = \begin{bmatrix} \frac{\partial L_0}{\partial Y_0} & \frac{\partial L_0}{\partial Y_1} \\ \frac{\partial L_1}{\partial Y_0} & \frac{\partial L_1}{\partial Y_1} \end{bmatrix}_{\hat{X}_0, \hat{X}_1, \hat{Y}_0, \hat{Y}_1} = \begin{bmatrix} \delta_0 + \kappa\tau_0 & -\kappa\tau_1 \\ -\kappa\tau_0 & \delta_1 + \kappa\tau_1 \end{bmatrix} \quad \{1.11\}$$

The inverse of **H** is calculated.

$$\mathbf{H}^{-1} = \frac{1}{\delta_0\delta_1 + \kappa(\delta_0\tau_1 + \delta_1\tau_0)} \begin{bmatrix} \delta_1 + \kappa\tau_1 & \kappa\tau_1 \\ \kappa\tau_0 & \delta_0 + \kappa\tau_0 \end{bmatrix} \quad \{1.12\}$$

The next generation matrix is then calculated by multiplying the matricies, **F** and $\mathbf{H}^{-1}$

$$\mathbf{K} = \mathbf{FH}^{-1}$$

$$= \frac{\beta}{\delta_0\delta_1 + \kappa(\delta_0\tau_1 + \delta_1\tau_0)} \begin{bmatrix} \hat{X}_0(1-z_0)/u_0 & 0 \\ 0 & \hat{X}_1(1-z_1)/u_1 \end{bmatrix} \begin{bmatrix} \delta_1 + \kappa\tau_1 & \kappa\tau_1 \\ \kappa\tau_0 & \delta_0 + \kappa\tau_0 \end{bmatrix}$$

{1.13}

Let $\varpi = \dfrac{\beta}{\delta_0\delta_1 + \kappa(\delta_0\tau_1 + \delta_1\tau_0)}$ be a dummy variable, defined for analytic tractability and then **K** can be rewritten as follows {1.14}

$$\mathbf{K} = \begin{bmatrix} \varpi \hat{X}_0(1-z_0)(\delta_1 + \kappa\tau_1)/u_0 & \varpi \hat{X}_0(1-z_0)\kappa\tau_1/u_0 \\ \varpi \hat{X}_1(1-z_1)\kappa\tau_0/u_1 & \varpi \hat{X}_1(1-z_1)(\delta_0 + \kappa\tau_0)/u_1 \end{bmatrix} \quad \{1.15\}$$

The effective reproductive number in the presence of therapy: *R*

Let λ be the eigenvalues of **K**

$$\begin{vmatrix} \varpi \hat{X}_0(1-z_0)(\delta_1 + \kappa\tau_1)/u_0 - \lambda & \varpi \hat{X}_0(1-z_0)\kappa\tau_1/u_0 \\ \varpi \hat{X}_1(1-z_1)\kappa\tau_0/u_1 & \varpi \hat{X}_1(1-z_1)(\delta_0 + \kappa\tau_0)/u_1 - \lambda \end{vmatrix} = 0 \quad \{1.16\}$$

$$\lambda^2 - \lambda\varpi\left(\hat{X}_0(1-z_0)(\delta_1 + \kappa\tau_1)/u_0 + \hat{X}_1(1-z_1)(\delta_0 + \kappa\tau_0)/u_1\right)$$
$$+ \varpi^2\hat{X}_0\hat{X}_1(1-z_1)(1-z_0)\left(\delta_0\delta_1 + \kappa(\delta_0\tau_1 + \delta_1\tau_0)\right)/u_0u_1 = 0 \quad \{1.17\}$$

$$\lambda = \frac{\varpi}{2}\left(\hat{X}_0(1-z_0)(\delta_1 + \kappa\tau_1)/u_0 + \hat{X}_1(1-z_1)(\delta_0 + \kappa\tau_0)/u_1\right)$$
$$\pm\frac{\varpi}{2}\sqrt{\left(\hat{X}_0(1-z_0)(\delta_1 + \kappa\tau_1)/u_0 + \hat{X}_1(1-z_1)(\delta_0 + \kappa\tau_0)/u_1\right)^2 - 4\hat{X}_0\hat{X}_1(1-z_1)(1-z_0)\left(\delta_0\delta_1 + \kappa(\delta_0\tau_1 + \delta_1\tau_0)\right)/u_0u_1}$$

{1.18}

The effective reproductive number, *R*, is then the largest eigenvalue of **K**

$$R = \frac{\beta}{2\left(\delta_0\delta_1 + \kappa(\delta_0\tau_1 + \delta_1\tau_0)\right)}\Bigg[\left(\hat{X}_0(1-z_0)(\delta_1 + \kappa\tau_1)/u_0 + \hat{X}_1(1-z_1)(\delta_0 + \kappa\tau_0)/u_1\right)$$
$$+\sqrt{\left(\hat{X}_0(1-z_0)(\delta_1 + \kappa\tau_1)/u_0 + \hat{X}_1(1-z_1)(\delta_0 + \kappa\tau_0)/u_1\right)^2 - 4\hat{X}_0\hat{X}_1(1-z_1)(1-z_0)\left(\delta_0\delta_1 + \kappa(\delta_0\tau_1 + \delta_1\tau_0)\right)/u_0u_1}\Bigg]$$

{1.19}

The basic reproductive number in the presence of treatment (antiretroviral therapy and trafficking therapy): *R*$_{0,T}$

To determine $R_{0,T}$ the effective reproductive number is evaluated at the uninfected equilibrium. Therefore, the number of susceptible cells in each compartment,

evaluated at the uninfected equilibrium ($\hat{X}_0 = \bar{X}_0 = \Lambda u_0/\alpha$ and $\hat{X}_1 = \bar{X}_1 = \Lambda u_1/\alpha$), are substituted into equation 1.19.

$$R_{0,T} = \frac{\beta\Lambda}{2\alpha\left(\delta_0\delta_1 + \kappa(\delta_0\tau_1 + \delta_1\tau_0)\right)}\Big[\left((1-z_0)(\delta_1 + \kappa\tau_1) + (1-z_1)(\delta_0 + \kappa\tau_0)\right) + \sqrt{\left((1-z_0)(\delta_1+\kappa\tau_1) + (1-z_1)(\delta_0+\kappa\tau_0)\right)^2 - 4(1-z_1)(1-z_0)\left(\delta_0\delta_1 + \kappa(\delta_0\tau_1+\delta_1\tau_0)\right)}\Big] \quad \{1.20\}$$

The basic reproductive number in the absence of therapy and assuming that the drug sanctuaries are not also immune sanctuaries: *R_0*

To determine R_0, we evaluate $R_{0,T}$ (equation 1.20) in the absence of antiretroviral therapy ($z_0 = z_1 = 0$) or trafficking therapy ($\kappa = 1$). We assume here that the drug sanctuaries are not immune sanctuaries, that is, the infected cell clearance rate is equal in both compartments ($\delta_0 = \delta_1$).

$$R_0 = \frac{\beta\Lambda}{\alpha\delta_1} \quad \{1.21\}$$

An expression for the threshold for ongoing replication in the presence of therapy in terms of $R_0, \kappa, \tau_0, \delta_0, \delta_1, z_0, z_1, u_0$

Substitute equations 1.21, 1.5 and $R_{0,T} > 1$ into equation 1.20

$$\kappa\tau_0 < \frac{\delta_1\left(\delta_1 R_0(1-z_0) - \delta_0\right)\left(R_0(1-z_1) - 1\right)}{u_0(1-u_0)^{-1}(\delta_1 R_0(1-z_0) - \delta_0) + \delta_1(R_0(1-z_1) - 1)} \quad \{1.22\}$$

The effective reproductive number if the drug sanctuaries represent only a small fraction of all CD4+ T-cells.

Data have revealed that CD4+ T-cells infected with HIV-1 in lymphoid tissue decline by several orders of magnitude following the onset of antiretroviral therapy (S2 and S3 references). This indicates that the population of CD4+ T-cells in the drug sanctuaries represents only a small fraction of the total population of CD4+ T-cells in the body. Thus, we sought to find an expression for the effective reproductive number, assuming the drug sanctuaries represent only a small fraction of the total body. This was achieved by evaluating equation 1.19 at the limit $u_0 \to 0$. Notice that

$\hat{X}_0 \sim o(u_0)$, therefore $\hat{X}_0/u_0 \sim o(1)$. Thus, we first defined $\Phi_0 = \hat{X}_0/u_0$ and substituted it into equation 1.19.

$$R = \frac{\beta}{2\left(\delta_0\delta_1 + \kappa(\delta_0\tau_1 + \delta_1\tau_0)\right)}\left[\left(\Phi_0(1-z_0)(\delta_1+\kappa\tau_1) + \hat{X}_1(1-z_1)(\delta_0+\kappa\tau_0)/u_1\right)\right.$$
$$\left. + \sqrt{\left(\Phi_0(1-z_0)(\delta_1+\kappa\tau_1) + \hat{X}_1(1-z_1)(\delta_0+\kappa\tau_0)/u_1\right)^2 - 4\Phi_0\hat{X}_1(1-z_1)(1-z_0)\left(\delta_0\delta_1+\kappa(\delta_0\tau_1+\delta_1\tau_0)\right)/u_1}\right]$$

{1.23}

Next, we let $u_0 = 0$ (therefore $\tau_1 = \tau_0 u_0/u_1 = 0$) and substitute this into equation 1.23.

$$R = \frac{\beta\Phi_0(1-z_0)}{(\delta_0+\kappa\tau_0)} \qquad \{1.24\}$$

To find an expression for *R* in terms of $\hat{X}_0$, substitute $\Phi_0 = \hat{X}_0/u_0$ into equation {1.24}.

$$R = \frac{\beta\hat{X}_0(1-z_0)}{u_0(\delta_0+\kappa\tau_0)} \qquad \{1.25\}$$

Notice that expression 1.25 is independent of the rate that infected cells in the main compartment traffic to the drug sanctuaries. This is because if the drug sanctuaries make up a very small fraction of the total body, infected cells that traffic from the drug sanctuaries to the main compartment will be highly diluted when they reach the main compartment, thus the rate that they flow back into the drug sanctuaries from the main compartment will be negligible.

The basic reproductive number if the drug sanctuaries represent only a small fraction of all CD4+ T-cells.

The number of susceptible cells in the drug sanctuaries, evaluated at the uninfected equilibrium ($\hat{X}_0 = \bar{X}_0 = \Lambda u_0/\alpha$), and expression 1.21 are substituted into equation 1.25.

$$R_{0,T} = \frac{R_0\delta_1(1-z_0)}{(\delta_0+\kappa\tau_0)} \qquad \{1.26\}$$

An expression for the threshold for ongoing replication if the drug sanctuaries represent only a small fraction of all CD4+ T-cells.

Substitute $u_0 = 0$ into equation 1.22

$$\kappa\tau_0 < \delta_1 R_0 (1 - z_0) - \delta_0 \qquad \{1.27\}$$

Equation 1.27 reveals that when the drug sanctuaries represent only a small fraction of all CD4+ T-cells in the body, the threshold for ongoing replication is governed by a linear relationship between the trafficking rate out of the drug sanctuaries and the treatment effectiveness in the drug sanctuaries (see Figure 2 of the main text).

References

S1. Diekmann O, Heesterbeek JA, Roberts MG. The construction of next-generation matrices for compartmental epidemic models. Journal of the Royal Society, Interface. 2010;7(47):873-85.

S2. Lorenzo-Redondo R, Fryer HR, Bedford T, Kim EY, Archer J, Kosakovsky Pond SL, et al. Persistent HIV-1 replication maintains the tissue reservoir during therapy. Nature. 2016;530(7588):51-6.

S3. Fletcher CV, Staskus K, Wietgrefe SW, Rothenberger M, Reilly C, Chipman JG, et al. Persistent HIV-1 replication is associated with lower antiretroviral drug concentrations in lymphatic tissues. Proc Natl Acad Sci U S A. 2014;111(6):2307-12.
