# Supplementary material for: Increased T cell trafficking as adjunct therapy for HIV-1
Source: PLoS Comput Biol. 2018 Mar 2;14(3):e1006028. doi: 10.1371/journal.pcbi.1006028 (PMC5864072; doi:10.1371/journal.pcbi.1006028)
Supplement: S2 Text — (PDF) [file pcbi.1006028.s002.pdf]

## S2 Text. The impact of a trafficking therapy on viral dynamics.

We aimed to investigate how the manner in which our modelled trafficking therapy redistributes susceptible and infected cells amongst the two compartments affects HIV-1 dynamics. In model 1, application of a trafficking therapy is assumed to increase the rate at which cells move to the opposing compartment by a factor, $\kappa$. In other words, the trafficking therapy increases both the rate at which cells leave the sanctuary and the rate at which cells enter it. This has two opposing effects on the amount of infection that persists in the drug sanctuaries, and therefore on the system as a whole. One effect is that an increase in trafficking moves infected cells away from the drug sanctuaries (where they can infect other cells), to the heavily treated main compartment (where they cannot infect other cells). This effect acts in favour of reducing infected cell numbers in the drug sanctuaries.

The second effect of trafficking therapy is that susceptible cells enter the drug sanctuary faster and the number of such cells ( $\hat{X}_0$ ) increases. Larger susceptible cell populations  lead to more infected cells in the drug sanctuaries so this effect acts to increase infected cell numbers in the drug sanctuaries. This is apparent from the fact that the effective reproductive number (equation 1.24) increases as $\hat{X}_0$ increases.

Which of these two opposing effects dominates depends upon the degree to which the trafficking rate is increased.  If trafficking rates are only slightly increased the growth in the number of susceptible cells dominates. Therefore the number of infected cells increases with small increases in the trafficking rate. At higher trafficking rates the removal of infected cells from drug sanctuaries dominates and the number of infected cells in the drug sanctuaries declines with increases in the trafficking rate. At sufficiently high trafficking rates (see threshold expression in equation 1.26) ongoing infection in drug sanctuaries is no longer sustainable.

A threshold condition defining the trafficking parameter, $\kappa$, at which the equilibrium number of infected cells in the sanctuaries starts to decline as $\kappa$ increases, was sought. For analytical tractability, we calculated this threshold under the scenario that cell clearance rates in each compartment are equal ($\delta_0 = \delta_1$) and ARV is perfectly

effective in the main compartment ($z_1 = 1$). First, the number of infected cells in the drug sanctuaries at the infected equilibrium (equation 2.3) was derived. For completeness the equilibrium numbers of susceptible and infected cells in each of the two compartments is also shown (equations 2.1-2.4).

$$\bar{X}_0 = \frac{u_0\delta_1(\delta_1 + \kappa\tau_0 + \kappa\tau_1)}{\beta(1-z_0)(\delta_1 + \kappa\tau_1)} \qquad \{2.1\}$$

$$\bar{X}_1 = \delta_1\left(\frac{\left(R_0(1-z_0)\alpha u_1 + \kappa\tau_0 u_0\right)(\delta_1 + \kappa\tau_1) + \kappa^2\tau_0^{\,2}u_0}{\beta(1-z_0)(\delta_1 + \kappa\tau_1)(\alpha + \kappa\tau_1)}\right) \qquad \{2.2\}$$

$$\bar{Y}_0 = \frac{\alpha u_0(u_1\alpha + \kappa\tau_0)}{\beta(1-z_0)(u_1\alpha + \kappa\tau_0 u_0)}\left(\frac{R_0(1-z_0)(u_1\delta_1 + \kappa\tau_0 u_0)}{(u_1\delta_1 + \kappa\tau_0)} - 1\right) \qquad \{2.3\}$$

$$\bar{Y}_1 = \frac{\alpha\kappa\tau_0}{\beta(1-z_0)(\alpha + \kappa\tau_1)}\left(\frac{R_0(1-z_0)(u_0\alpha + \kappa\tau_1)}{(\delta_1 + \kappa\tau_0 + \kappa\tau_1)} - \frac{u_0(\alpha + \kappa\tau_0 + \kappa\tau_1)}{(\delta_1 + \kappa\tau_1)}\right) \qquad \{2.4\}$$

Second, the partial derivative with respect to $\kappa$, of the equilibrium number of infected cells in the drug sanctuaries was derived and set to zero ($\partial\bar{Y}_0 / \partial\kappa = 0$). The threshold condition on $\kappa$, above which trafficking therapy reduces the burden of HIV-1, is given as:

$$\kappa > \frac{(1-u_0)}{\tau_0}\left(\frac{-\alpha\delta_1 + \sqrt{\delta\alpha R_0(\alpha-\delta_1)(1-z_0)\left(R_0(\alpha-\delta_1)(1-z_0)u_0 - \alpha + \delta_1 u_0\right)}}{\alpha - R_0(\alpha-\delta_1)(1-z_0)u_0}\right) \qquad \{2.5\}$$

Model simulations confirm that if the trafficking rate is increased to a rate that is below the threshold value (S2a Figure), infected cell counts marginally increase in both the drug sanctuaries and the main compartment. It is noteworthy that if the trafficking rate is reduced by a trafficking therapy ($\kappa = 0.5$ in S2b Figure), infected cell counts marginally decrease.

a)

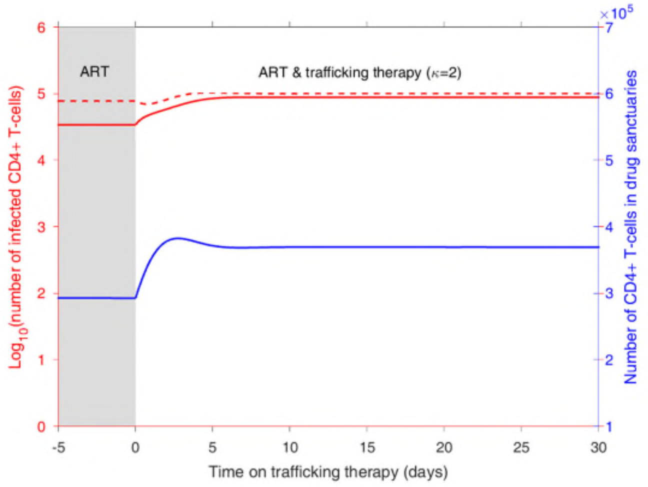


b)

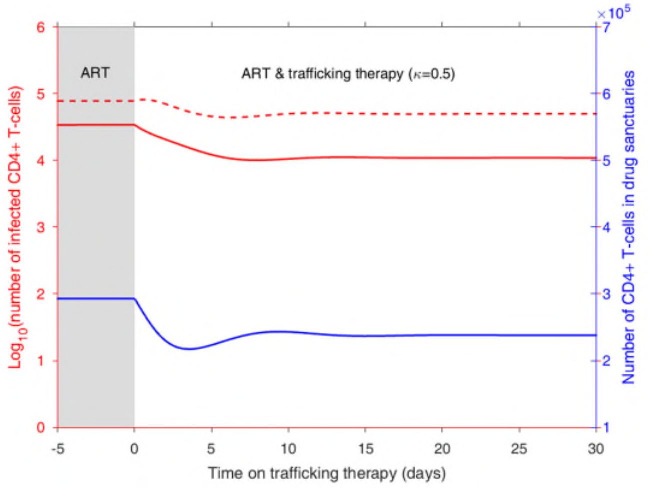


**S2 Figure. Viral dynamics resulting from trafficking therapy are also influenced by changes in susceptible cell numbers in drug sanctuaries.**

These figures show model predictions of the impact of changing the rate of trafficking therapy on infected cell numbers in the drug sanctuaries (solid red lines) and main compartment (dashed red lines). In each figure, prior to time 0 (grey shaded area), the host is only taking antiretroviral drugs. At this stage, the effectiveness of antiretroviral therapy is high in the main compartment of the body ( $z_1 = 0.97$), but lower in the drug sanctuaries ( $z_0 = 0.6$). Furthermore, the rate that cells traffic between compartments (governed by parameter $\tau_0 = 0.5$ day$^{-1}$) and the rate that infected cells are cleared from the drug sanctuaries ($\delta_0 = \delta_1 = 1$), are both slow enough to allow ongoing cycles of replication to persist in the drug sanctuaries. At time 0 a trafficking therapy is applied which changes the rate that cells move between compartments. In a) the trafficking rate is only slightly increased ($\kappa = 2$), resulting in a small increase in the number of infected cells in both the drug sanctuaries and the main compartment. This results from an increased supply of susceptible cells to the drug sanctuaries. In b) a therapy which decreases the trafficking rate is applied ($\kappa = 0.5$) and a small decline in infected cell numbers is predicted because of a decline in the supply of susceptible cells to the drug sanctuaries.

To further explore the role that trafficking, in relation to its effect on population sizes, has on infection dynamics, we have adapted model 1 to ensure that trafficking therapy does not directly affect the total number of cells in the sanctuaries (Model 2, equations 2.6-2.9). In this adaptation, the total additional flow rate of cells into the sanctuaries (defined as σ day$^{-1}$) is the same as the additional flow rate out of the sanctuaries. For simplicity, this model ignores the impact of immune sanctuaries and immune therapy; thus infected cell clearance rates in each compartment equal $\delta$ day$^{-1}$. To investigate this model we ran model simulations at varying total additional flow rates due to trafficking therapy, σ day$^{-1}$ (see S3 Figure). They show that if trafficking therapy does not directly affect total cell numbers in the different spatial compartments, the number of infected cells in the drug sanctuaries always declines with therapy that increases the trafficking rate. Furthermore there remains a threshold pace of mixing at which infection is no longer sustainable.

**Model 2: ordinary differential equation for a model in which trafficking therapy does not directly affect population sizes in each of the compartments**

$$dX_0/dt = \Lambda u_0 - \beta X_0(1-z_0)Y_0/u_0 - (\alpha+\tau_0)X_0 + \tau_1 X_1 - \sigma X_0/N_0 + \sigma X_1/N_1 \qquad \{2.6\}$$

$$dX_1/dt = \Lambda u_1 - \beta X_1(1-z_1)Y_1/u_1 - (\alpha+\tau_1)X_1 + \tau_0 X_0 - \sigma X_1/N_1 + \sigma X_0/N_0 \qquad \{2.7\}$$

$$dY_0/dt = \beta X_0(1-z_0)Y_0/u_0 - (\delta+\tau_0)Y_0 + \tau_1 Y_1 - \sigma Y_0/N_0 + \sigma Y_1/N_1 \qquad \{2.8\}$$

$$dY_1/dt = \beta X_1(1-z_1)Y_1/u_1 - (\delta+\tau_1)Y_1 + \tau_0 Y_0 - \sigma Y_1/N_1 + \sigma Y_0/N_0 \qquad \{2.9\}$$

.

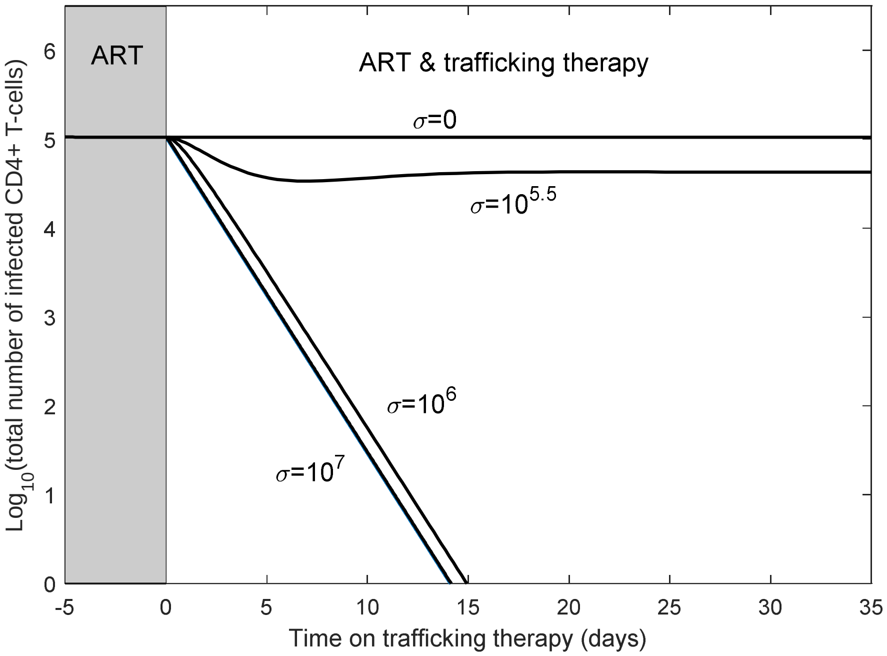


**S3 Figure. Trafficking therapy that does not have an impact upon the total number of cells in each compartment, always reduces the total number of infected cells.**

This figure shows model predictions of trafficking therapy that does not impact the number of cells in each compartment, on infection dynamics. Prior to time 0 (grey shaded area), the host is only taking antiretroviral drugs. At this stage, the effectiveness of antiretroviral therapy is high in the main compartment of the body ( $z_1 = 0.97$), but lower in the drug sanctuaries ( $z_0 = 0.6$). Furthermore, the rate that cells traffic between compartments (governed by parameter $\tau_0 = 0.5$ day$^{-1}$) and the rate that infected cells are cleared from the drug sanctuaries ($\delta = 1$), are both slow enough to allow ongoing cycles of replication to persist in the drug sanctuaries. At time 0 a trafficking therapy is applied which changes the rate that cells move between compartments. Different trafficking rates, governed by the total flow rate between compartments ($\sigma$ day$^{-1}$) are explored. This reveals that trafficking therapy that doesn't impact cell numbers in each of the compartments always reduces the total number of infected cells. Furthermore, there remains a threshold condition on the trafficking therapy parameter above which replication is no longer sustainable.
