# Supplementary material for: Increased T cell trafficking as adjunct therapy for HIV-1
Source: PLoS Comput Biol. 2018 Mar 2;14(3):e1006028. doi: 10.1371/journal.pcbi.1006028 (PMC5864072; doi:10.1371/journal.pcbi.1006028)
Supplement: S3 Text — (PDF) [file pcbi.1006028.s003.pdf]

S3 Text. Model adaptations show that reducing the inflow of CD4+ T-cells into, or increasing the outflow of CD4+ T-cells out of drug sanctuaries can stop persistent replication

We sought to investigate how independently changing either the rate that CD4+ T-cells flow into or out of drug sanctuaries impacts viral dynamics. We therefore created Model 3 which is the same as model 1, except that trafficking therapy affects the rates that cells flow into and out of drug sanctuaries independently. The outflow rate is changed by a factor κ_0 and the inflow rate by a factor κ_1. The effect of immune sanctuaries and immune therapy that increases the clearance rate of infected cells from drug sanctuaries was excluded from this model. The infected cell clearance rate in both compartments is δ day^{-1}. Numerical simulations (not shown) confirm that immune therapy would work in synergy with trafficking therapy in a similar manner to that described for model 1.

Model 3 equations

$$\frac{dX_0}{dt} = \Lambda u_0 - \beta X_0 Y_0 (1-z_0)/u_0 - \alpha X_0 - \kappa_0 \tau_0 X_0 + \kappa_1 \tau_1 X_1 \qquad \{3.1\}$$

$$\frac{dX_1}{dt} = \Lambda u_1 - \beta X_1 Y_1 (1-z_1)/u_1 - \alpha X_1 - \kappa_1 \tau_1 X_1 + \kappa_0 \tau_0 X_0 \qquad \{3.2\}$$

$$\frac{dY_0}{dt} = \beta X_0 Y_0 (1-z_0)/u_0 - \delta Y_0 - \kappa_0 \tau_0 Y_0 + \kappa_1 \tau_1 Y_1 \qquad \{3.3\}$$

$$\frac{dY_1}{dt} = \beta X_1 Y_1 (1-z_1)/u_1 - \delta Y_1 - \kappa_1 \tau_1 Y_1 + \kappa_0 \tau_0 Y_0 \qquad \{3.4\}$$

$$\tau_0 u_0 = \tau_1 u_1 \qquad \{3.5\}$$

Using the next generation approach described in S1 Text we calculated a threshold for ongoing replication for this model.

$$\kappa_0 \tau_0 \left(1 - R_0(1-z_1)\right) - \kappa_1 \tau_1 \left(R_0(1-z_0) - 1\right) < \delta \left(R_0(1-z_0) - 1\right)\left(1 - R_0(1-z_1)\right) \qquad \{3.6\}$$

Notice that the condition for ongoing replication in the drug sanctuaries, but not in the main compartment of the body in the absence of trafficking therapy requires that $R_0(1-z_1) < 1$ and $R_0(1-z_0) > 1$. Therefore, expression 3.6 reveals that a trafficking

therapy that either increases the outflow rate of cells from (larger κ_0) or decreases the inflow rate of cells to (smaller κ_1) the drug sanctuaries would be capable of halting ongoing replication in drug sanctuaries. This finding is confirmed by numerical simulations (S4 Figure).

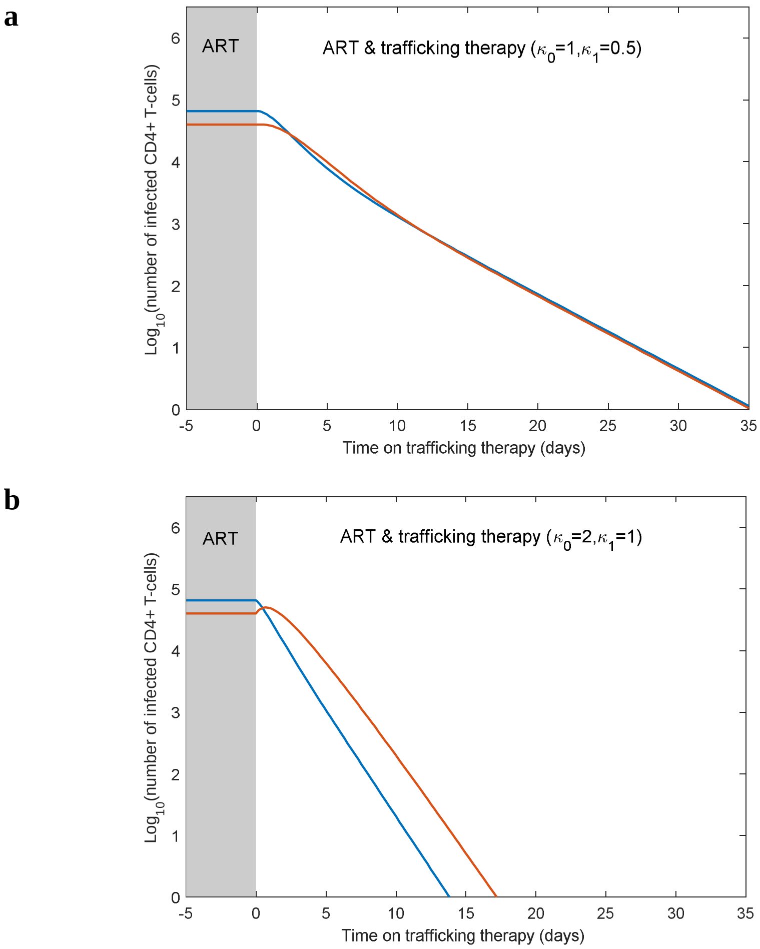

S4 Figure. Trafficking therapy that reduces the flow of CD4+ T-cells into drug sanctuaries or increases their outflow from drug sanctuaries can stop persistent replication. This figure shows the impact of therapy that independently changes the rate that CD4+ T-cells traffic into and out of drug sanctuaries. Infected cell numbers in the drug sanctuaries (blue line) and main compartment (red line) are plotted. Prior to time 0 (grey shaded area), the host is only taking antiretroviral drugs. At this stage, the effectiveness of antiretroviral therapy is high in the main compartment of the body ($z_1 = 0.97$), but lower in the drug sanctuaries ($z_0 = 0.6$), and trafficking between compartments is slow enough to allow ongoing replication to persist in the drug sanctuaries ($\tau_0 = 0.5$ day^{-1}). At time 0, a trafficking therapy is additionally applied. In a) trafficking therapy reduces the rate that cells flow into the drug sanctuaries by a

factor of 2 ($\kappa_1 = 0.5$) but has no impact on the rate they flow out ($\kappa_0 = 1$). This intervention stops persistent replication. In b) trafficking therapy increases the rate that cells flow out of drug sanctuaries by a factor of 2 ($\kappa_0 = 2$), but has no impact upon the rate they flow in ($\kappa_1 = 1$). This intervention also stops persistent replication
