# Supplementary material for: Increased T cell trafficking as adjunct therapy for HIV-1
Source: PLoS Comput Biol. 2018 Mar 2;14(3):e1006028. doi: 10.1371/journal.pcbi.1006028 (PMC5864072; doi:10.1371/journal.pcbi.1006028)
Supplement: S4 Text — (PDF) [file pcbi.1006028.s004.pdf]

S4 Text. Infection via free virus does not influence the findings of our study.

To understand how infection via free virus influences the findings of our study, we began by developing a version of our binary spatial model in which infection of CD4+T-cells occurs only via free virions (Model 4). Here, infectious free virions (*V*) are assumed to mix homogenously throughout the system and their spatial location does not need to be tracked. Infectious virions are produced by infected cells at rate *p* day^{-1} and they are cleared at rate *c* day^{-1}. For analytic tractability the infected cell clearance rate in both compartments is assumed to equal δ day^{-1}.

Model 4: A model with homogenous mixing of free virus

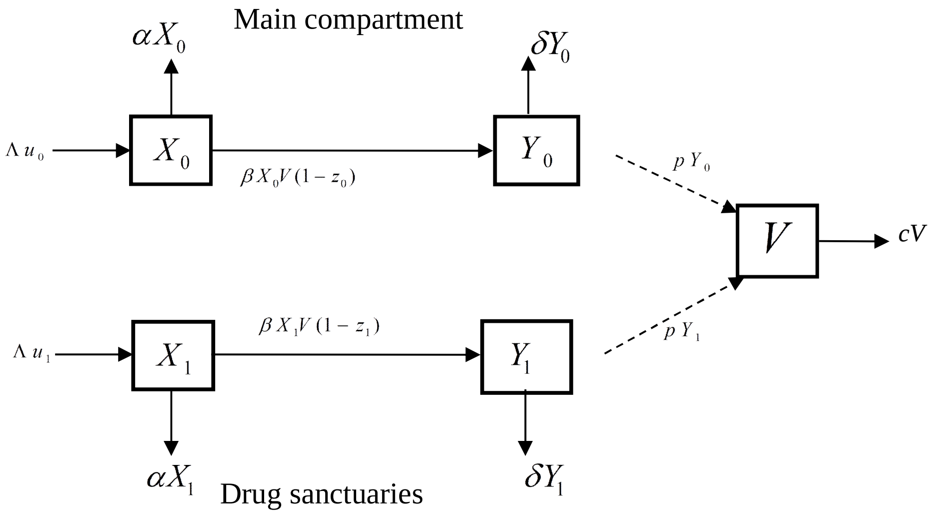

Model 4 equations

$$\frac{dX_0}{dt} = \Lambda u_0 - \beta X_0 V(1-z_0) - \alpha X_0 \qquad \{4.1\}$$

$$\frac{dX_1}{dt} = \Lambda u_1 - \beta X_1 V(1-z_1) - \alpha X_1 \qquad \{4.2\}$$

$$\frac{dY_0}{dt} = \beta X_0 V(1-z_0) - \delta Y_0 \qquad \{4.3\}$$

$$\frac{dY_1}{dt} = \beta X_1 V(1-z_1) - \delta Y_1 \qquad \{4.4\}$$

$$\frac{dV}{dt} = p\left(Y_0 + Y_1\right) - cV \qquad \{4.5\}$$

We sought to understand whether under these assumptions, stable ongoing cycles of replication could occur in drug sanctuaries when replication is suppressed in the main compartment of the body. Therefore, we calculated expressions for the basic reproductive number in the absence (R_0) and the presence ($R_{0,T}$) of therapy, as well the threshold for ongoing replication.

As described in S1 Text we derived the next generation matrix across. In this scenario, however, it is calculated across both infected cells and free virus (equation 4.6).

$$\mathbf{K} = \begin{bmatrix} 0 & 0 & \beta \hat{X}_0(1-z_0)c^{-1} \\ 0 & 0 & \beta \hat{X}_1(1-z_1)c^{-1} \\ p\delta^{-1} & p\delta^{-1} & 0 \end{bmatrix} \tag{4.6}$$

In this scenario, the effective reproductive number that relates only to cells (or only to virions) – that is, the number of new cell infections (virions) caused by one cell infection (virion) is equal to the square of the largest eigenvalue of the next generation matrix (S4 reference). Using the principals described in S1 Text we can derive:

The basic reproductive number in the absence of therapy: R_0

$$R_0 = \frac{\beta p \Lambda}{c \alpha \delta} \tag{4.7}$$

The basic reproductive number in the presence of therapy: $R_{0,T}$

$$R_{0,T} = R_0\left(u_0(1-z_0)+u_1(1-z_1)\right) \tag{4.8}$$

Expression for *V* at the infected, untreated equilibrium ($\tilde{V}$)

By setting the left hand side of equations 4.1-4.5 to equal 0, and setting $z_0 = z_1 = 0$ we derived an expression (dependent upon R_0) for free virions ($\tilde{V}$) at the infected, untreated equilibrium.

$$\tilde{V} = \left(R_0 - 1\right)\frac{\alpha}{\beta} \tag{4.9}$$

Expression including *V* at the infected, treated equilibrium

By setting the left hand side of equations 4.1-4.5 to equal 0, we derived an expression (dependent upon R_0 and $R_{0,T}$) for free virions ($\breve{V}$) at the infected, treated equilibrium.

$$(R_{0,T}-1)=\left(\frac{\beta\breve{V}}{\alpha}\right)^2(1-z_0)(1-z_1)+\frac{\beta\breve{V}}{\alpha}\big((1-z_0)+(1-z_1)-R_0(1-z_0)(1-z_1)\big) \qquad \{4.10\}$$

Expression 4.9 was substituted into equation 4.10 to derive an expression dependent upon $\tilde{V}$

$$(R_{0,T}-1)=\left(\frac{\breve{V}}{\tilde{V}}\right)^2(R_0-1)^2(1-z_0)(1-z_1)+\left(\frac{\breve{V}}{\tilde{V}}\right)(R_0-1)\big((1-z_0)+(1-z_1)-R_0(1-z_0)(1-z_1)\big)$$

{4.11}

Using this expression it is possible to understand conditions under which a large decline in viral load could be observed following the onset of therapy.

Note that when $\breve{V}/\tilde{V}\to 0$ then $R_{0,T}\to 1$

Therefore, it shows that a large decline in viral load can only be observed when the basic reproductive number under therapy is very close to 1. The instability of this criteria means that it is not consistent with a theory of *stable* ongoing replication in drug sanctuaries. Although trafficking of T-cells between compartments is not included in this model, the same effects are observed when trafficking is included.

To model how infection by free virus affects our results, we therefore developed Model 5, which includes spatial heterogeneity to infectious free virus infection (see below). This model is able to capture the observed large decline of free virus numbers in a setting of stable ongoing replication in drug sanctuaries.

Model 5 is similar to model 1, however, infected cells within each spatial compartment are assumed to produce infectious free virions into the same compartment at rate p day^{-1}. Virions can only infect cells within the same compartment; however there is also some trafficking of free virions between

compartments. Virions from compartment *i* move to the other compartment at rate $\tilde{\tau}_i$ day^{-1} in the absence of a trafficking therapy. In the presence of a trafficking therapy the rate is increased by a factor $\tilde{\kappa}$. Virions are cleared at rate c day^{-1}. For analytic tractability the infected cell clearance rate in both compartments is assumed to equal δ day^{-1}. However, numerical simulations with different cell clearance rates (not shown) confirm that immune therapy would work in synergy with trafficking therapy in a similar manner to that described for model 1.

Model 5: A model with spatial heterogeneity for free virus

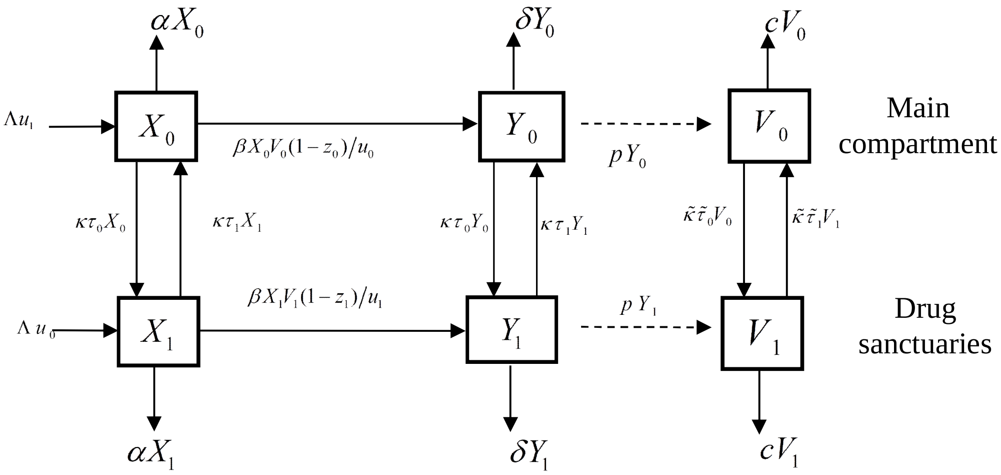

Model 5 equations

$$\frac{dX_0}{dt} = \Lambda u_0 - \beta X_0 V_0 (1-z_0)/u_0 - (\alpha + \kappa\tau_0) X_0 + \kappa\tau_1 X_1 \quad \{4.12\}$$

$$\frac{dX_1}{dt} = \Lambda u_1 - \beta X_1 V_1 (1-z_1)/u_1 - (\alpha + \kappa\tau_1) X_1 + \kappa\tau_0 X_0 \quad \{4.13\}$$

$$\frac{dY_0}{dt} = \beta X_0 V_0 (1-z_0)/u_0 - (\delta + \kappa\tau_0) Y_0 + \kappa\tau_1 Y_1 \quad \{4.14\}$$

$$\frac{dY_1}{dt} = \beta X_1 V_1 (1-z_1)/u_1 - (\delta + \kappa\tau_1) Y_1 + \kappa\tau_0 Y_0 \quad \{4.15\}$$

$$\frac{dV_0}{dt} = pY_0 - (c + \tilde{\kappa}\tilde{\tau}_0) V_0 + \tilde{\kappa}\tilde{\tau}_1 V_1 \quad \{4.16\}$$

$$\frac{dV_1}{dt} = pY_1 - (c + \tilde{\kappa}\tilde{\tau}_1) V_1 + \tilde{\kappa}\tilde{\tau}_0 V_0 \quad \{4.17\}$$

$$\tau_0 u_0 = \tau_1 u_1 \quad \{4.18\}$$

$$\tilde{\tau}_0 u_0 = \tilde{\tau}_1 u_1 \quad \{4.19\}$$

We sought to find an analytic expression for the threshold condition for ongoing replication in drug sanctuaries by first deriving an expression for the basic reproductive number in the presence ($R_{0,T}$) and absence (R_0) of therapy. As described in more detail in S1 Text, this was derived by finding the next generation matrix (**K**).

As described in S1 Text we derived the next generation matrix across. In this scenario, however, it is calculated across both infected cells and free virus (equation 4.20).

$$\mathbf{K}=\begin{bmatrix} 0 & 0 & -\beta\hat{X}_0(1-z_0)(c+\tilde{\kappa}\tilde{\tau}_1)/c\tilde{\varsigma}u_0 & -\beta\hat{X}_0(1-z_0)\tilde{\kappa}\tilde{\tau}_1/c\tilde{\varsigma}u_0 \\ 0 & 0 & -\beta\hat{X}_1(1-z_1)\tilde{\kappa}\tilde{\tau}_0/c\tilde{\varsigma}u_1 & -\beta\hat{X}_1(1-z_1)(c+\tilde{\kappa}\tilde{\tau}_0)/c\tilde{\varsigma}u_1 \\ -p(\delta+\kappa\tau_1)/\delta\varsigma & -p\kappa\tau_1/\delta\varsigma & 0 & 0 \\ -p\kappa\tau_0/\delta\varsigma & -p(\delta+\kappa\tau_0)/\delta\varsigma & 0 & 0 \end{bmatrix}$$

{4.20}

Where ς and $\tilde{\varsigma}$ are dummy variables, defined for analytic tractability as follows:

$$\varsigma=\delta+\kappa\tau_1+\kappa\tau_0 \qquad \{4.21\}$$

$$\tilde{\varsigma}=c+\tilde{\kappa}\tilde{\tau}_1+\tilde{\kappa}\tilde{\tau}_0 \qquad \{4.22\}$$

In this scenario, the effective reproductive number that relates only to cells (or only to virions) – that is, the number of new cell infections (virions) caused by one cell infection (virion) is equal to the square of the largest eigenvalue of the next generation matrix (S4 reference). Using the principals described in S1 Text we can derive:

The basic reproductive number in the absence of therapy: *R*₀

$$R_0=\frac{\beta\,p\Lambda}{\alpha\,\delta\,c} \qquad \{4.23\}$$

The basic reproductive number in the presence of therapy: $R_{0,T}$

$$R_{0,T}=\frac{R_0}{2\varsigma\tilde{\varsigma}}\Big[(1-z_0)\big(c(\delta+\kappa\tau_1)+\tilde{\kappa}\tilde{\tau}_1\varsigma\big)+(1-z_1)\big(c(\delta+\kappa\tau_0)+\tilde{\kappa}\tilde{\tau}_0\varsigma\big)$$
$$+\sqrt{\Big((1-z_0)\big(c(\delta+\kappa\tau_1)+\tilde{\kappa}\tilde{\tau}_1\varsigma\big)+(1-z_1)\big(c(\delta+\kappa\tau_0)+\tilde{\kappa}\tilde{\tau}_0\varsigma\big)\Big)^2-4(1-z_0)(1-z_1)c\delta}\,\Big]$$

{4.24}

An expression defining the threshold for ongoing replication

$$R_0^{\,2}(1-z_0)(1-z_1)c\delta - R_0\left((1-z_0)\left(c(\delta+\kappa\tau_1)+\tilde{\kappa}\tilde{\tau}_1\varsigma\right)+(1-z_1)\left(c(\delta+\kappa\tau_0)+\tilde{\kappa}\tilde{\tau}_0\varsigma\right)\right)+\varsigma\tilde{\varsigma}<0$$

{4.25}

An expression for the threshold for ongoing replication when the drug sanctuaries represent a small fraction of the total body size

$$(\delta+\kappa\tau_0)(c+\tilde{\kappa}\tilde{\tau}_0)<(1-z_0)R_0\delta c \qquad \{4.26\}$$

This representation demonstrates that ongoing replication in drug sanctuaries is dependent not only upon treatment effectiveness in the drug sanctuaries (z_0) being sufficiently low, but also upon trafficking of both cells (at rate $\kappa\tau_0$) and free virions (at rate $\tilde{\kappa}\tilde{\tau}_0$) out of the drug sanctuaries being sufficiently slow. Thus, this model, in which all infection is via free virions, suggests that a trafficking therapy designed to increase trafficking of either CD4 T cells, free virions, or both has the potential eliminate viral replication. The threshold condition would be expected to be dependent upon the ratio of new infections that typically result from each of the two routes.

Reference

S4. Roberts MG, Heesterbeek JA. A new method for estimating the effort required to control an infectious disease. Proceedings Biological sciences. 2003;270(1522):1359-64.
