# Supplementary material for: Increased T cell trafficking as adjunct therapy for HIV-1
Source: PLoS Comput Biol. 2018 Mar 2;14(3):e1006028. doi: 10.1371/journal.pcbi.1006028 (PMC5864072; doi:10.1371/journal.pcbi.1006028)
Supplement: S5 Text — (PDF) [file pcbi.1006028.s005.pdf]

S5 Text. If cells carry their treatment status with them as they migrate, there is still a threshold on the pace of CD4+ T-cell trafficking that can support ongoing replication in drug sanctuaries

In model 1 it is assumed that therapy acts at the level of the cells' environment. Thus, it is implicitly assumed that cells that become treated within one compartment will not remain treated if they move to another compartment. In reality, antiretrovirals such as reverse transcriptase inhibitors can be absorbed into cells and retained, at least temporarily, as the cells move around the body. Drug concentration-dependent diffusion out of the cell and the natural decay rate of the drug will influence the time spent in a treated state.

Here we investigate whether our assumption that treatment acts at the level of the cells' environment affects our finding that ongoing replication in drug sanctuaries is governed by a threshold condition on the pace of CD4+ T-cell trafficking. We developed an adapted version of our model in which treated cells can retain their treated status as they move from one spatial compartment to another. In this model (Model 6), the number of treated (W_i) and untreated cells (X_i) in each spatial compartment is modelled explicitly. Treatment penetrating compartment *i* acts to convert untreated cells into treated cells at rate π_i day^{-1}. Treated cells can also revert to their untreated status at rate ϕ day^{-1}. For analytic tractability the infected cell clearance rate in both compartments is assumed to equal δ day^{-1}.However, numerical simulations (not shown) confirm that immune therapy would work in synergy with trafficking therapy in a similar manner to that described for model 1. Model 6 is presented below.

Model 6:

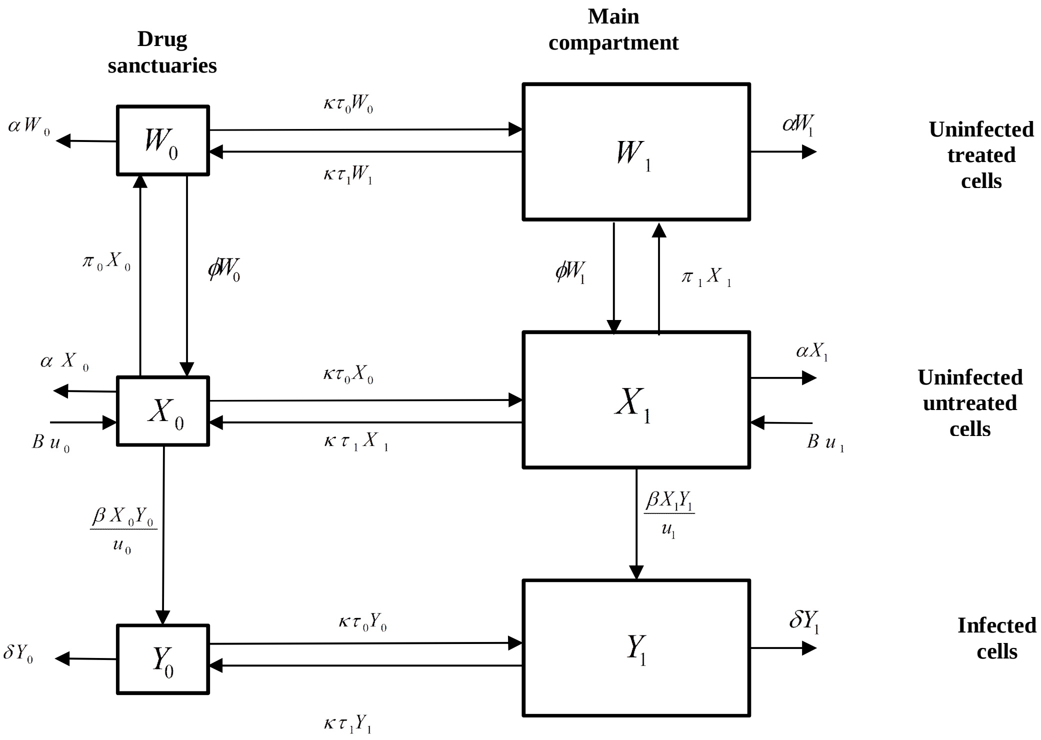

Model 6 equations

$$\frac{dW_0}{dt} = \pi_0 X_0 - (\alpha + \phi + \kappa\tau_0)W_0 + \kappa\tau_1 W_1 \qquad \{5.1\}$$

$$\frac{dW_1}{dt} = \pi_1 X_1 - (\alpha_1 + \phi + \kappa\tau_1)W_1 + \kappa\tau_0 W_0 \qquad \{5.2\}$$

$$\frac{dX_0}{dt} = \Lambda u_0 - \beta X_0 Y_0 / u_0 + \phi W_0 - (\pi_0 + \alpha + \kappa\tau_0) X_0 + \kappa\tau_1 X_1 \qquad \{5.3\}$$

$$\frac{dX_1}{dt} = \Lambda u_1 - \beta X_1 Y_1 / u_1 + \phi W_1 - (\pi_1 + \alpha + \kappa\tau_1) X_1 + \kappa\tau_0 X_0 \qquad \{5.4\}$$

$$\frac{dY_0}{dt} = \beta X_0 Y_0 / u_0 - (\delta + \kappa\tau_0) Y_0 + \kappa\tau_1 Y_1 \qquad \{5.5\}$$

$$\frac{dY_1}{dt} = \beta X_1 Y_1 / u_1 - (\delta + \kappa\tau_1) Y_1 + \kappa\tau_0 Y_0 \qquad \{5.6\}$$

$$\tau_0 u_0 = \tau_1 u_1 \qquad \{5.7\}$$

Using the next generation approach described in more detail in S1 Text, we calculated an expression for the basic reproductive number ($R_{0,T}$ in the presence of therapy and R_0 in the absence of therapy) and a threshold for ongoing replication.

The basic reproductive number in the absence of therapy: *R*₀

Derived by setting $\pi_0 = \pi_1 = 0$ and $\kappa = 1$ {5.8}

$$R_0 = \frac{\beta\Lambda}{\delta\alpha} \quad \{5.9\}$$

The basic reproductive number in the presence of therapy: $R_{0,T}$

$$R_{0,T} = \frac{R_0(\alpha+\phi)}{2(\delta+\kappa\tau_1+\kappa\tau_0)}\left((\pi_0+\alpha+\phi)^{-1}(\delta+\kappa\tau_1)+(\pi_1+\alpha+\phi)^{-1}(\delta+\kappa\tau_0)\right)$$
$$+\frac{R_0(\alpha+\phi)}{2(\delta+\kappa\tau_1+\kappa\tau_0)}\sqrt{\left((\pi_0+\alpha+\phi)^{-1}(\delta+\kappa\tau_1)+(\pi_1+\alpha+\phi)^{-1}(\delta+\kappa\tau_0)\right)^2-4(\pi_1+\alpha+\phi)^{-1}(\pi_0+\alpha+\phi)^{-1}\delta(\delta+\kappa\tau_1+\kappa\tau_0)}$$

{5.10}

An expression for the threshold for ongoing replication

$$\left(\frac{(\delta+\kappa\tau_1+\kappa\tau_0)(\pi_0+\alpha+\phi)}{R_0(\alpha+\phi)}-(\delta+\kappa\tau_1)\right)\left(\frac{(\delta+\kappa\tau_1+\kappa\tau_0)(\pi_1+\alpha+\phi)}{R_0(\alpha+\phi)}-(\delta+\kappa\tau_0)\right)<\kappa^2\tau_1\tau_0$$

{5.11}

Threshold for ongoing replication when the drug sanctuaries represent only a small fraction of all CD4+ T-cells, $u_0 \approx 0$

$$\kappa\tau_0 < \delta\left(\frac{R_0(\alpha+\phi)}{(\pi_0+\alpha+\phi)}-1\right) \quad \{5.12\}$$

This equation demonstrates that ongoing replication in drug sanctuaries is not only dependent upon the uptake of ART within the sanctuaries (π_0) being sufficiently slow, but also dependent upon the trafficking of CD4+ T-cells between the drug sanctuaries and the main compartment being sufficiently slow.
