# Supplementary material for: Increased T cell trafficking as adjunct therapy for HIV-1
Source: PLoS Comput Biol. 2018 Mar 2;14(3):e1006028. doi: 10.1371/journal.pcbi.1006028 (PMC5864072; doi:10.1371/journal.pcbi.1006028)
Supplement: S6 Text — (PDF) [file pcbi.1006028.s006.pdf]

S6 Text. α4β7 antibody therapy and a functional cure for HIV-1

It has been demonstrated that antibodies that bind alpha 4 beta7, when taken alongside antiretroviral therapy, lead to sustained improved virological control of simian immunodeficiency virus (SIV) (S5 reference) in macaques. These drugs have now been approved for clinical trial use in HIV-1 infected individuals. The precise mechanism by which the improvement in macaques was achieved is unknown. What is clear is that when these antibodies are used in combination with antiretroviral therapy, the combination is better than antiretroviral therapy alone. One possible explanation for this effect is that the binding of antibodies to virions leads directly to opsonization of the virions by phagocytes (S6 reference). A second explanation is that $\alpha_4\beta_7$ antibody therapy trafficked infected CD4+ T-cells away from the gut into regions where ART is more effective. A third possible explanation is that upon antibody binding, the propensity for virions to home to the high endothelial venules (HEVs) where they can easily infect passing target cells is reduced (S6 reference). Each of these explanations is consistent the theory that persistent rounds of infection are maintained in lymphatic tissue 'drug sanctuaries' in the ART-treated control monkeys. The latter two are also with a theory that trafficking manipulation was involved in helping to control infection (see S3 Text).

It remains an open question how any treatment given only temporarily – and not expected to deal with the latent reservoir, as indeed $\alpha_4\beta_7$ antibodies would not – could achieve the post-interruption control that was reported. Post-treatment HIV-1 controllers from the VISCONTI study (S7 reference) provide a proof of principle for viral control in the absence of viral eradication. A 'functional cure' for HIV-1 that results in indefinite control of virus remains an important goal of current research efforts. Periodic regimens whereby patients are regularly tested and resume therapy if necessary, could be useful in an alternative circumstance where a functional cure can achieve long-term, but not indefinite, control of HIV-1 infection.

References

S5. Byrareddy SN, Arthos J, Cicala C, Villinger F, Ortiz KT, Little D, et al. Sustained virologic control in SIV+ macaques after antiretroviral and alpha4beta7 antibody therapy. Science. 2016;354(6309):197-202.

S6. Guzzo C, Ichikawa D, Park C, Phillips D, Liu Q, Zhang P, et al. Virion incorporation of integrin alpha4beta7 facilitates HIV-1 infection and intestinal homing. Science immunology. 2017;2(11).

S7. Saez-Cirion A, Bacchus C, Hocqueloux L, Avettand-Fenoel V, Girault I, Lecuroux C, et al. Post-treatment HIV-1 controllers with a long-term virological remission after the interruption of early initiated antiretroviral therapy ANRS VISCONTI Study. PLoS pathogens. 2013;9(3):e1003211.
