# Supplementary material for: Increased T cell trafficking as adjunct therapy for HIV-1
Source: PLoS Comput Biol. 2018 Mar 2;14(3):e1006028. doi: 10.1371/journal.pcbi.1006028 (PMC5864072; doi:10.1371/journal.pcbi.1006028)
Supplement: S7 Text — (PDF) [file pcbi.1006028.s007.pdf]

S7 Text. Manipulating the trafficking of CD4 T-cells to germinal centres.

By considering the pathways involved in CD4 T-cell trafficking to the germinal centres in lymph nodes, we can speculate on avenues for therapeutic manipulation of them. We note that, as these pathways are central to the immune response they would need to be very carefully manipulated.

CD4 T-cells respond to sphingosine-1-phosphate (S1P) to egress the follicles (References S8-S10). It may be possible to encourage CD4 T-cells to egress the follicles by enhancing the levels of S1P though manipulation of the S1P lyases that normally degrade S1P. Follicular helper T-cells (Tfh), however, are preferentially retained in the follicles because of their low expression of sphingosine-1-phosphate receptor 1 (S1PR1). This means that they would not respond to changes in S1P expression. One possible approach to promoting the egress of Tfh cells would therefore involve therapeutically promoting the expression of S1PR1 (potentially through transient blockade of phosphatidylinositol-3-kinase (PI3K) (S11 and S12 references).

Sphingosine-1-phosphate receptor 2 (S1PR2) and the orphan receptor, P2Y receptor family member 8 (P2RY8) are also involved in confining Tfh cells to germinal centres (S13 and S14 references). It is possible that antagonism of S1PR2 would promote some release of some Tfh from the germinal environment, though their access to circulation would likely still be limited by their low S1PR1 expression. S1PR2 is expressed on many cell types such as endothelium, so there would be risks of off-target effects.

References

S8. Hunter MC, Teijeira A, Halin C. T Cell Trafficking through Lymphatic Vessels. Frontiers in immunology. 2016;7:613.

S9. Cyster JG. Chemokines, sphingosine-1-phosphate, and cell migration in secondary lymphoid organs. Annual review of immunology. 2005;23:127-59.

S10. Baeyens A, Fang V, Chen C, Schwab SR. Exit Strategies: S1P Signaling and T Cell Migration. Trends in immunology. 2015;36(12):778-87.

S11. Bai A, Hu H, Yeung M, Chen J. Kruppel-like factor 2 controls T cell trafficking by activating L-selectin (CD62L) and sphingosine-1-phosphate receptor 1 transcription. Journal of immunology. 2007;178(12):7632-9.

S12. Huddleson JP, Ahmad N, Lingrel JB. Up-regulation of the KLF2 transcription factor by fluid shear stress requires nucleolin. The Journal of biological chemistry. 2006;281(22):15121-8.

S13. Moriyama S, Takahashi N, Green JA, Hori S, Kubo M, Cyster JG, et al. Sphingosine-1-phosphate receptor 2 is critical for follicular helper T cell retention in germinal centers. The Journal of experimental medicine. 2014;211(7):1297-305.

S14. Muppidi JR, Lu E, Cyster JG. The G protein-coupled receptor P2RY8 and follicular dendritic cells promote germinal center confinement of B cells, whereas S1PR3 can contribute to their dissemination. The Journal of experimental medicine. 2015;212(13):2213-22.
