# Supplementary material for: Increased T cell trafficking as adjunct therapy for HIV-1
Source: PLoS Comput Biol. 2018 Mar 2;14(3):e1006028. doi: 10.1371/journal.pcbi.1006028 (PMC5864072; doi:10.1371/journal.pcbi.1006028)
Supplement: S1 Fig — (PDF) [file pcbi.1006028.s008.pdf]

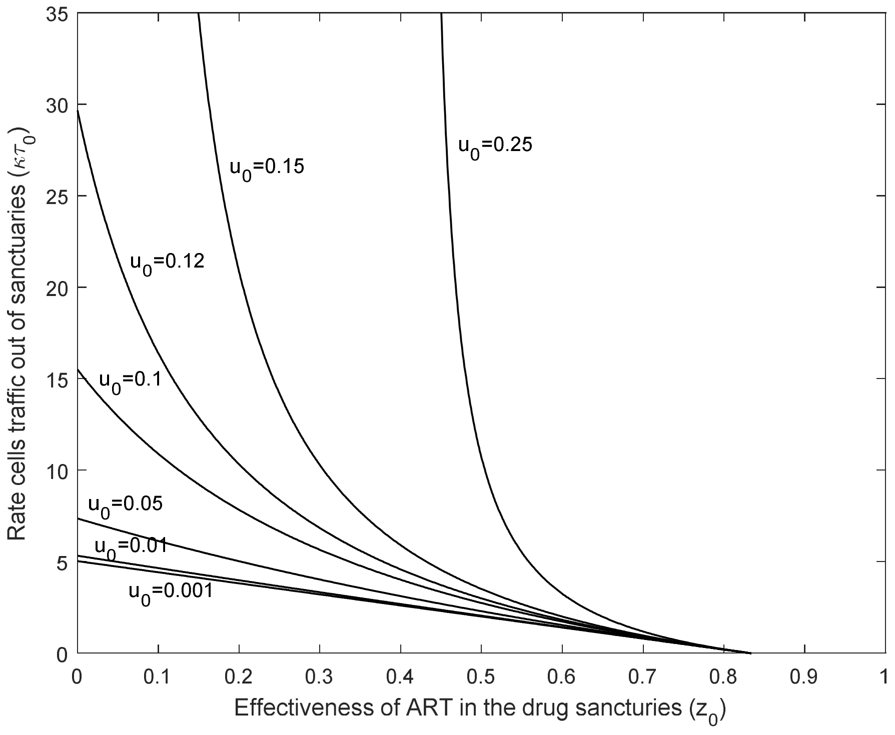

S1 Figure. The threshold for ongoing replication is dependent upon several factors including the rate of CD4+ T-cell trafficking and the size of the drug sanctuaries. This figure shows the relationship between the rate that CD4+ T-cells traffic out of drug sanctuaries ($\kappa\tau_0$) and the effectiveness of ART in the drug sanctuaries (z_0) at the threshold for ongoing replication. This relationship is shown for different sized drug sanctuaries (governed by u_0 = the fraction of CD4 T-cells in the drug sanctuaries). For small sanctuaries, the relationship is independent of u_0. In this scenario there is a negative relationship between the effectiveness of ART in the drug sanctuaries and the rate of cell trafficking between compartments at the threshold. For larger sanctuaries, the threshold pace of trafficking also increases with u_0. As the size of the sanctuaries increases further, at low ART efficacies in the drug sanctuaries (z_0), replication is ongoing, irrespective of pace of trafficking. In this scenario, increasing the pace of trafficking of CD4 T-cells would not be expected to contribute to viral clearance in the drug sanctuaries.
