# Supplementary material for: Increased T cell trafficking as adjunct therapy for HIV-1
Source: PLoS Comput Biol. 2018 Mar 2;14(3):e1006028. doi: 10.1371/journal.pcbi.1006028 (PMC5864072; doi:10.1371/journal.pcbi.1006028)
Supplement: S1 Table — (PDF) [file pcbi.1006028.s009.pdf]

S1 Table 1. Parameters for model 1.

Parameter	Description	Value used	Reference
α	Per cell clearance rate of susceptible cells (day^{-1})	0.01	S15 and S16 references.
δ_i	Per cell clearance rate of infected cells in compartment *i* (day^{-1})	$\delta_1 = 1$<br><br>$\delta_0 = 0.5$ (Fig 5a, 5b)<br>$\delta_0 = 1$ (Fig 5c, 5d)	S17 reference.<br><br>Different values were used to explore the impact of immune therapy that can reverse impaired cell clearance rates in the drug sanctuaries.
u_i	The fraction of the body in compartment *i*	$u_0 = 3.69 \times 10^{-5}$<br>$u_1 = 1 - u_0$	S2 reference.
z_i	Effectiveness in compartment *i* of the antiretroviral therapy in preventing the infection of cells.	$z_0 = 0.6$<br><br>$z_1 = 0.97$	Assumed to represent drug sanctuaries<br><br>To model a main compartment where drugs penetrate effectively.
β	Transmission coefficient (day^{-1})	4.3×10^{-10}	Estimated to fix the basic reproductive number (R_0=6) (S18 reference).
Λ	Birth rate of uninfected cells (day^{-1})	1.4×10^{8}	Estimated by fitting the model to infected cell data prior to ART (S3 reference).
κ	Factor increase in the trafficking rate caused by trafficking therapy	1 (Fig 4c)<br>3.5 (Fig 4b, 4d)<br>5 (Fig 4a)	These values were chosen to demonstrate the different impact upon viral dynamics of the addition of a trafficking therapy
τ_i	Per cell rate of traffic of cells from compartment *i* to the other compartment in the absence trafficking therapy (day^{-1})	$\tau_0 = 0.5$<br><br>$\tau_1 = \tau_0 u_0 / u_1$	τ_0 is assumed to be one half the clearance rate of infected cells (δ) in the absence of a trafficking therapy<br><br>τ_1 is scaled to account for the compartment size

References

S2. Lorenzo-Redondo R, Fryer HR, Bedford T, Kim EY, Archer J, Kosakovsky Pond SL, et al. Persistent HIV-1 replication maintains the tissue reservoir during therapy. Nature. 2016;530(7588):51-6.

S3. Fletcher CV, Staskus K, Wietgrefe SW, Rothenberger M, Reilly C, Chipman JG, et al. Persistent HIV-1 replication is associated with lower antiretroviral drug concentrations in lymphatic tissues. Proc Natl Acad Sci U S A. 2014;111(6):2307-12.
S15. De Boer RJ, Perelson AS. Quantifying T lymphocyte turnover. Journal of theoretical biology. 2013;327:45-87.

S16. Stafford MA, Corey L, Cao Y, Daar ES, Ho DD, Perelson AS. Modeling plasma virus concentration during primary HIV infection. Journal of theoretical biology. 2000;203(3):285-301.

S17. Markowitz M, Louie M, Hurley A, Sun E, Di Mascio M, Perelson AS, et al. A novel antiviral intervention results in more accurate assessment of human immunodeficiency virus type 1 replication dynamics and T-cell decay in vivo. Journal of virology. 2003;77(8):5037-8.

S18. Ribeiro RM, Qin L, Chavez LL, Li D, Self SG, Perelson AS. Estimation of the initial viral growth rate and basic reproductive number during acute HIV-1 infection. Journal of virology. 2010;84(12):6096-102.
